# Dataset of the infrared spectrometry, gas chromatography-mass spectrometry analysis and nuclear magnetic resonance spectroscopy of the polysaccharides from *C*. *militaris*

**DOI:** 10.1016/j.dib.2019.104126

**Published:** 2019-06-11

**Authors:** Min Liu, Lin Fu, Xiaoning Jia, Jin Wang, Xiaoqian Yang, Bin Xia, Ting Li, Fahui Li, Shoudong Guo

**Affiliations:** a Shandong First Medical University & Shandong Academy of Medical Sciences, Taian 271000, China; b Institute of Lipid Metabolism and Atherosclerosis, School of Pharmacy, Weifang Medical University, Weifang 261042, China

**Keywords:** Polysaccharide, Mushroom, GC-MS, FTIR spectrum, Methylation analysis, NMR, FTIR, fourier transform infrared spectrometry, GC-MS, gas chromatography-mass spectrometry, NMR, nuclear magnetic resonance spectroscopy

## Abstract

The data presented in this article describe characteristics of the polysaccharides, designated as CM1 and CMS, isolated from the fruiting body of *C. militaris*. Fourier transform infrared spectrometry analysis was used to identify the basic characteristics of the polysaccharides and the completeness of methylation. Gas chromatography-tandem mass spectrometry and nuclear magnetic resonance spectroscopy were carried out to reveal the glycosidic linkages of CM1 and CMS. Further interpretation and discussion could be found at our research article entitled “Structural characterisation and cholesterol efflux improving capacity of the novel polysaccharides from *Cordyceps militaris*” (Hu et al., 2019; https://doi.org/10.1016/j.ijbiomac.2019.03.078) [1].

**Table:** Specifications Table

Subject area	*Chemistry*
More specific subject area	*Analytical chemistry*
Type of data	*Figure, Table*
How data was acquired	*FTIR was recorded on a Nicolet iS5 spectrometer, GC-MS was performed using a 5977A MSD instrument equipped with a DB 225 fused silica capillary column (0.25 mm* *×* *30 m), NMR was carried out using a JEOL JNM-ECP 600 MHz spectrometer*
Data format	*Raw and Analyzed*
Experimental factors	*Purified polysaccharides CM1 and CMS were obtained by a Q-Sepharose*^*TM*^*Fast Flow column with distilled water as the eluent and then further separated by a Sephacryl S200HR column.*
Experimental features	*Tablets for IR analysis were prepared by mixing dried sample with milled KBr under an infrared lamp; For GC-MS analysis, methylated CM1 and CMS were hydrolysed and then acetylated before analysis.*
Data source location	*School of Pharmacy, Weifang Medical University, Weifang, China.*
Data accessibility	*Data are presented in this article. Raw data is available as supplementary file.*
Related research article	*S.M. Hu, J. Wang, F.H. Li, P.B. Hou, J.Y. Yin, Z.X. Yang, X.Q. Yang, T. Li, B. Xia, G.H. Zhou, M. Liu, W.G. Song, S.D. Guo, Structural characterisation and cholesterol efflux improving capacity of the novel polysaccharides from Cordyceps militaris. International Journal of Biological Macromolecules, 2019, 131: 264–272*[1].

**Table:** 

**Value of the data** • FTIR data are helpful for clarifying the characteristics of the polysaccharides and the completeness of the methylation. • GC-MS and NMR data are useful information for elucidating the glycosidic linkages of CM1 and CMS. • The data benefit the chemical researchers, and especially carbohydrate researchers focusing on structural characteristics of polysaccharide. • The data are helpful for structural elucidation of other polysaccharides, and especially the one with similar structural characteristics.

## 1 Data

*Cordyceps* species are valuable food source and famous traditional medicinal mushrooms. Recently, there is an increasing tendency to use *C. militaris* as a substitute for *C. sinensis*. The polysaccharide CM1 and CMS was obtained from the fruiting body of *C. militaris* by a Q-Sepharose™ Fast Flow column with distilled water as the eluent and then further separated by a Sephacryl S200HR column (see the descriptions in Ref. [1]).

**Fig. 1:**
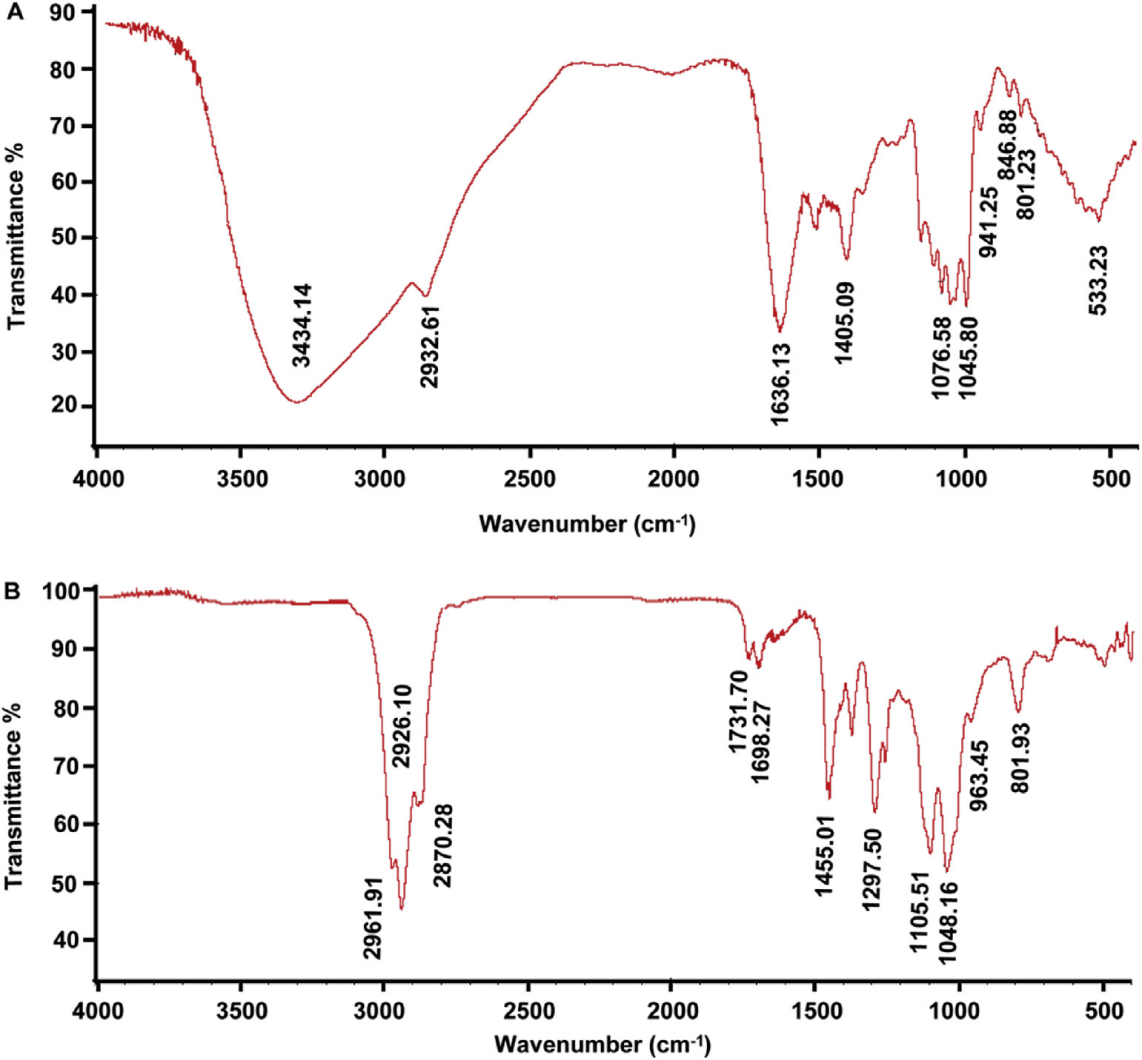
IR spectra of the heteropolysaccharide CM1 from *C. militaris* before and after methylation. (A), IR spectrum of CM1; (B), IR spectrum of CM1 after methylation.

As shown in the FTIR spectrum of CM1 (Fig. 1A, raw data Supplementary Fig. 1), the broad and strong signal at 3434.14 cm^−1^ was due to the stretching vibration of the hydroxyl group (-OH), and the signal at 1076.58 cm^−1^ originated from the bending vibrations of the O–H bond. The bands at 2932.61 cm^−1^ and 1405.09 cm^−1^ were assigned to the stretching vibrations and bending vibrations of alkyl groups (–CH_2_– and –CH_3_), respectively. The signals in the range of 1045.80–1090.39 cm^−1^ were attributed to the stretching vibrations of C–O–C and C–O–H. The characteristic absorption at 801.23 originated from mannose, and the peak at 846.88 cm^−1^ was attributed to the α-anomeric configuration of the glycosyl units. The absorption at 941.25 cm^−1^ revealed the presence of a furanoid ring [2], [3], [4]. After complete methylation (Fig. 1B, raw data Supplementary Fig. 2), the signal at 3434.14 cm^−1^ representing the stretching vibration of O–H disappeared, and the stretching vibration of alkyl groups (–CH_2_– and –CH_3_) at 2926.10 cm^−1^ significantly increased. Fig. 1B revealed the completeness of methylation.

**Fig. 2:**
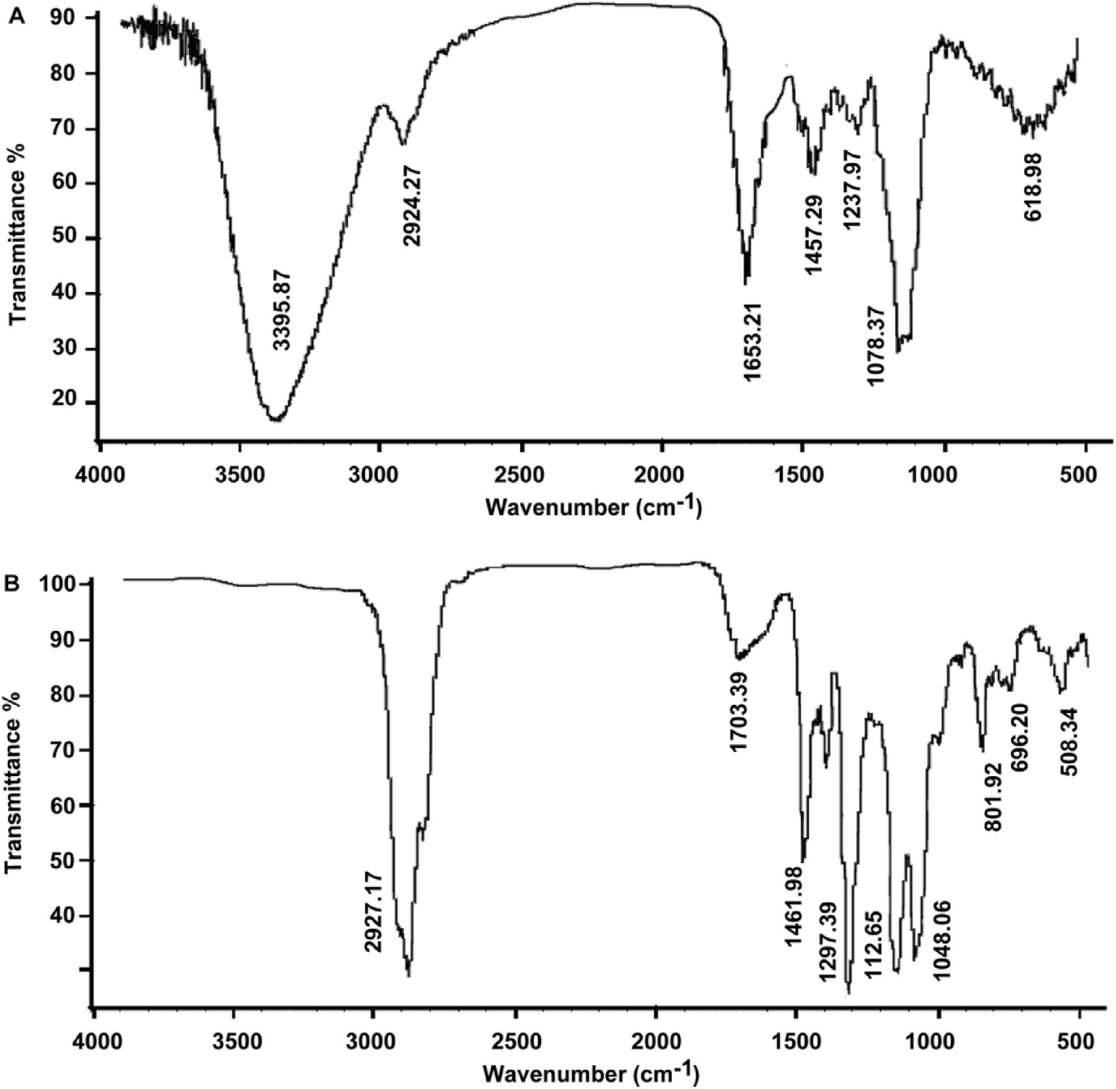
IR spectra of the heteropolysaccharide CMS from *C. militaris* before and after methylation. (A), IR spectrum of CMS; (B), IR spectrum of CMS after methylation.

As shown in the IR spectrum of CMS (Fig. 2A, raw data Supplementary Fig. 3), the broad and strong signal at 3395.87 cm^−1^ was due to the stretching vibration of the hydroxyl group (-OH), and the signal at 1078.37 cm^−1^ originated from the bending vibrations of the O–H bond. The bands at 2924.27 cm^−1^ were assigned to the stretching vibrations of alkyl groups (–CH_2_–). The signals around 1050 cm^−1^ were attributed to the stretching vibrations of C–O–C and C–O–H. Fig. 2B (raw data Supplementary Fig. 4) revealed the completeness of methylation.

**Fig. 3:**
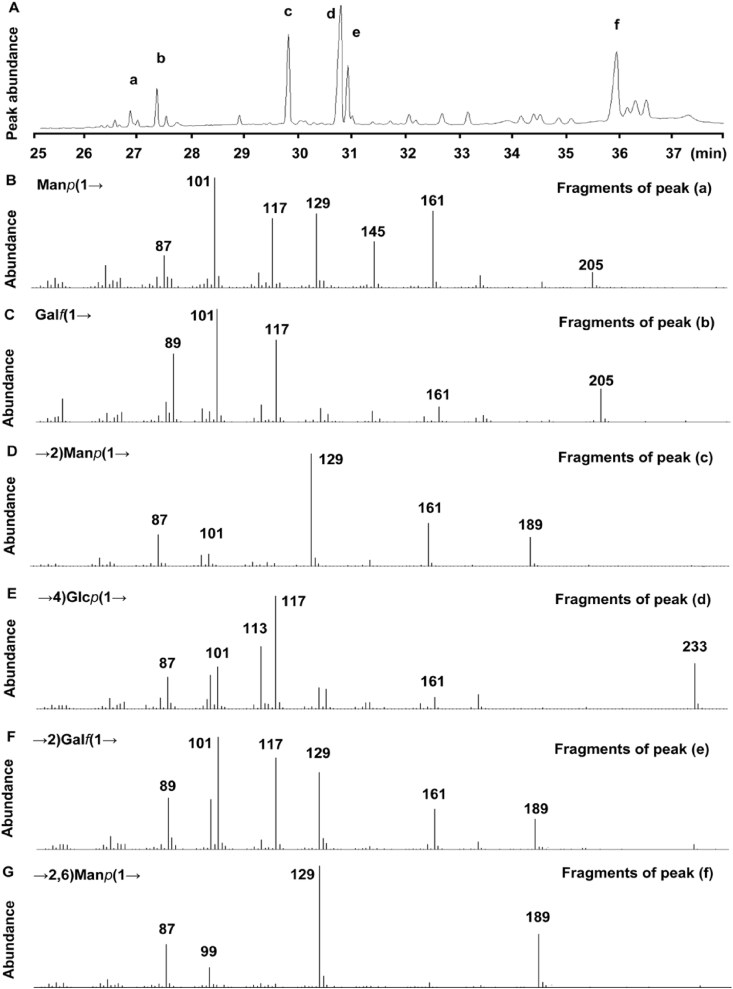
The total ion chromatogram of CM1 and their characteristic fragments as measured by GC-MS. (A), the characteristic GC-MS spectrum of CM1 after complete methylation; (B), characteristic fragments of the glycosyl Man*p*(1→; (C), characteristic fragments of the glycosyl Gal*f*(1→; (D), characteristic fragments of the glycosyl →2)Man*p*(1→; (E), characteristic fragments of the glycosyl →4)Glc*p*(1→; (F), characteristic fragments of the glycosyl →2)Gal*f*(1→; (G) characteristic fragments of the glycosyl →2,6)Man*p*(1→.

**Fig. 4:**
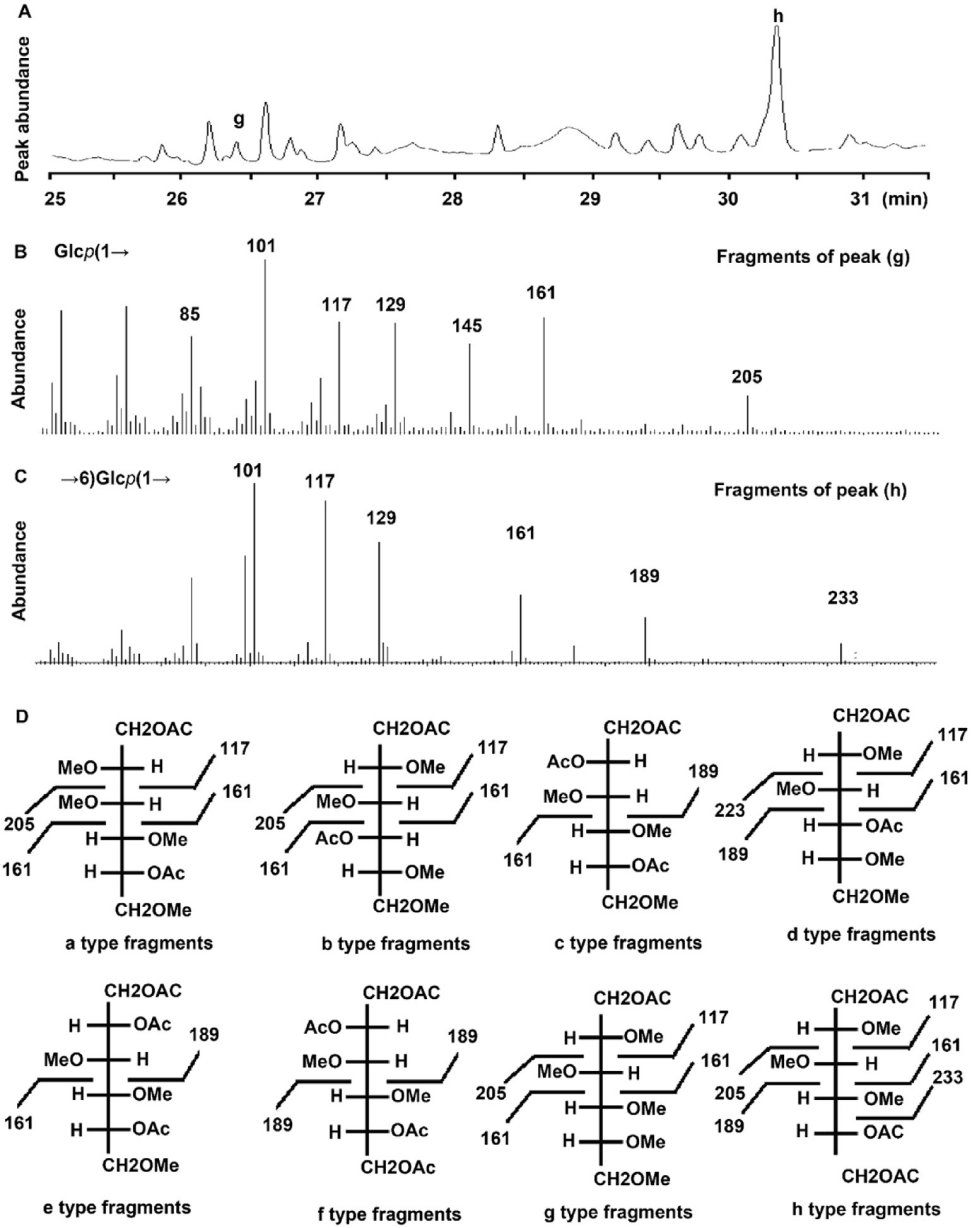
The total ion chromatogram of CMS and their characteristic fragments as measured by GC-MS. (A), the characteristic GC-MS spectrum of CMS after complete methylation; (B), characteristic fragments of the glycosyl Glc*p*(1→; (C), characteristic fragments of the glycosyl →6)Glc*p*(1→; (D), interpretation of the characteristic fragment patterns of a-h as listed in Fig. 3, Fig. 4.

In the data, the glycosyl linkage patterns were revealed using GC-MS after complete methylation (Fig. 3, Fig. 4 and Table 1, raw data Supplementary Fig. 5A and B). As for CM1, the non-reducing end Man*p*(1→ had characteristic fragments of 87.0, 101.0, 117.0, 129.0, 145.0 and 161.0; Gal*f*(1→ had the characteristic fragments of 89.0, 101.0, 117.0, 161.0 and 205.0; →2)Man*p*(1→ linked residue had the characteristic fragments of 87.0, 101.0, 129.0, 161.0 and 189.0; →4)Glc*p*(1→ linked residue had the characteristic fragments of 87.0, 101.0, 113.0, 117.0, 161.0 and 233.0; →2)Gal*f*(1→ linked residue had the characteristic fragments of 89.0, 117.0, 129.0, 161.0 and 189.0; →2,6)Man*p*(1→ linked residue had the characteristic fragments of 87.0, 99.0, 129.0 and 189.0. As for CMS, the non-reducing end Glc*p*(1→ had characteristic fragments of 101.0, 117.0, 129.0, 145.0, 161.0 and 205.0; →6)Glc*p*(1→ linked residue had the characteristic fragments of 101.0, 117.0, 129.0, 161.0, 189.0, and 233.0. As for CMS, only minor Glc*p*(1→ and major →6)Glc*p*(1→ linked residues were detected.

**Table 1:** Methylation analysis of CM1 and CMS.

Methylation residues	Linkage patterns	Molar ratio		Major mass fragments (m/z)
		CM1	CMS	
2,3,4,6-Me_4_-Man*p*	Man*p*(1→	1.0	/	87, 101, 117, 129, 145, 161
2,3,5,6-Me_4_-Gal*f*	Gal*f*(1→	2.5	/	89, 101, 117, 161, 205
3,4,6-Me_3_-Man*p*	→2)Man*p*(1→	1.5	/	87, 101, 129, 161, 189
2,3,6-Me_3_-Glc*p*	→4)Glc*p*(1→	4.0	/	87, 101, 113, 117, 161, 233
3,5,6-Me_3_-Gal*f*	→2)Gal*f*(1→	2.3	/	89, 117, 129, 161, 189
3,4-Me_2_-Man*p*	→2,6)Man*p*(1→	3.1	/	87, 99, 129, 189
2,3,4,6,-Me_4_-Glc*p*	Glc*p*(1→	/	1.0	101, 117, 129, 145, 161, 205
2,3,4- Me_3_-Glc*p*	→6)Glc*p*(1→	/	81.3	101, 117, 129, 161, 189, 233

Table 2 listed the major carbohydrate NMR data of CM1 and CMS, see the descriptions in Ref. [1]. As for CMS, we presume there may containing minor phenol compounds, which need to be characterized in the future (for 2D-NMR raw data please see Supplementary Figs. 6–13).

**Table 2:** ^1^H and^13^C chemical shifts for CM1 and CMS.

Sugar residues	H1/C1	H2/C2	H3/C3	H4/C4	H5/C5	H6/C6	OCH3
A (CM1)	5.15	4.06	3.90	3.74	3.74	4.18,4.23	
α-D-Man*p*(1→	102.10	70.91	71.91	68.51	74.04	59.42	
B (CM1)	5.06	4.05	3.88	3.69	3.74	4.18,4.23	
→2)α-D-Man*p*(1→	100.68	78.38	70.24	68.51	74.04	59.42	
C (CM1)	5.01	4.02	4.05	3.79	3.69	3.54,3.58	
β-D-Gal*f*(1→	106.25	81.12	76.28	81.79	72.54	62.33	
D (CM1)	4.99	4.05	4.10	3.90	3.73	3.53,3.58	
→2)β-D-Gal*f*(1→	106.25	87.04	76.10	81.79	70.24	62.83	
E (CM1)	4.78	3.94	3.89	3.84	3.79	3.68,4.04	3.08
→2,6)α-D-Man*p*(1→	99.30	78.38	70.91	68.51	70.56	65.96	54.10
F (CM1)	4.29	3.41	3.56	3.78	3.70	4.10,3.93	
→4)β-D-Glc*p*(1→	103.34	72.54	75.21	81.12	75.20	61.11	
A (CMS)	4.88	3.45	/	/	/	3.70,3.62	
α-D-Glc*p*(1→	98.12	71.25	72.3	68.56	72.81	63.22	
B (CMS)	4.78	3.30	3.38	3.54	3.74	3.96,3.72	
→6)β-D-Glc*p*	94.06	73.76	76.28	70.67	74.86	69.13	
C (CMS)	4.38	3.19	3.36	3.48	3.62	4.07,3.78	
→6)β-D-Glc*p*(1→	103.28	73.62	75.87	70.24	75.18	69.13	

## 2 Experimental design, materials and methods

### 2.1 Materials

Dimethyl sulfoxide, trifluoroacetic acid, CH_2_Cl_2_, CH_3_I, and KBr were products of Sigma-Aldrich. Double-deionized water was produced using a Milli-Q gradient system from Millipore (Bedford, MA). The remaining reagents used were of analytical grade.

### 2.2 Methylation analysis

Methylation analysis was performed according to the modified Hakomori method [2]. Briefly, 2.0 mg of polysaccharide was dissolved in 1.0 mL of dimethyl sulfoxide, and then, 100 mg of anhydrous sodium hydride was added. The mixture was stirred at room temperature for 1 h. One milliliter of CH_3_I was added to the mixture, followed by stirring for 2 h. Finally, the reaction was terminated with the addition of distilled water, and the resulting solution was extracted with CH_2_Cl_2_. The extract was washed with distilled water and evaporated to dryness. The permethylated polysaccharide was hydrolysed with 1.0 mL of 2.0 M trifluoroacetic acid at 110 °C for 6 h. The resulting hydrolysates were reduced with NaBH_4_, and acetylated with acetic anhydride.

### 2.3 GC-MS analysis

The methylated alditol acetates were analyzed by gas chromatography–mass spectrometry (GC–MS) using a 5977A MSD instrument equipped with a DB 225 fused silica capillary column (0.25 mm × 30 m) (Agilent Technologies Co. Ltd., USA) using a temperature gradient of 100–220 °C with heating at a rate of 5 °C/min and the maintenance of a temperature of 220 °C for 15 min [3]. The peaks on the chromatogram were identified on the basis of their retention times and mass fragmentation patterns.

### 2.4 FTIR spectroscopy analysis

For Fourier-transform infrared spectrometry (FTIR) analysis, the polysaccharide was mixed with KBr powder, ground and pressed into 1 mm pellets for measurement in the frequency range of 4000–500 cm^−1^. The FTIR spectrum of the polysaccharide was recorded on a Nicolet iS5 spectrometer [4], [5], [6].

### 2.5 FTIR spectroscopy analysis

Nuclear magnetic resonance (NMR) spectroscopy analyses including ^1^H-NMR, ^13^C-NMR, distortionless enhancement by polarization transfer spectroscopy (DEPT), ^1^H–^1^H correlated spectroscopy (COSY), ^1^H–^13^C heteronuclear multiple quantum coherence spectroscopy (HMQC), ^1^H–^13^C heteronuclear multiple bond correlation spectroscopy (HMBC) and nuclear Overhauser effect spectroscopy (NOESY) were performed using a JEOL JNM-ECP 600 MHz spectrometer [5], [7].
