# Supplementary figures and images for: Dataset of the infrared spectrometry, gas chromatography-mass spectrometry analysis and nuclear magnetic resonance spectroscopy of the polysaccharides from C. militaris
Source: Data Brief. 2019 Jun 11;25:104126. doi: 10.1016/j.dib.2019.104126 (PMC6595413; doi:10.1016/j.dib.2019.104126)

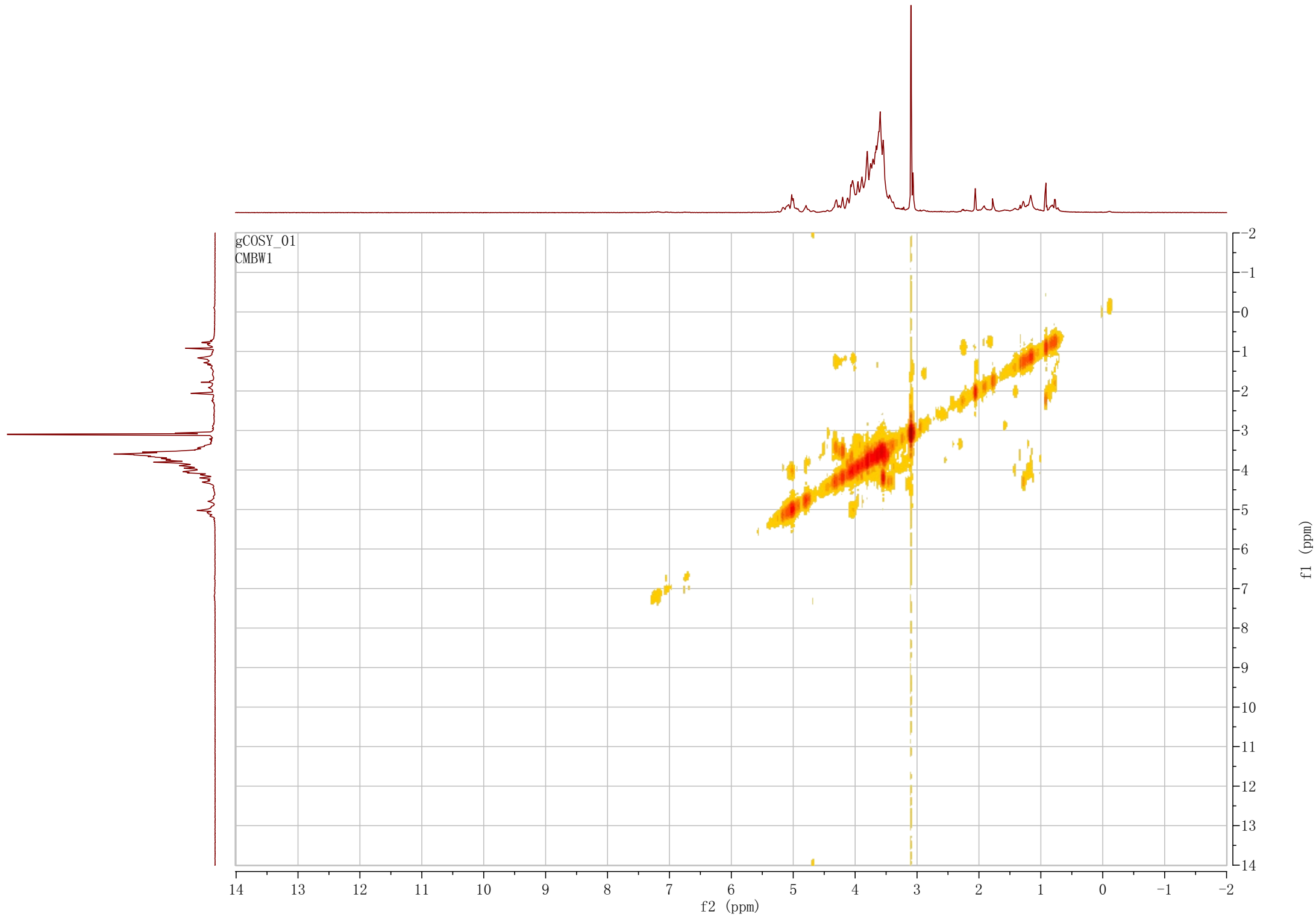

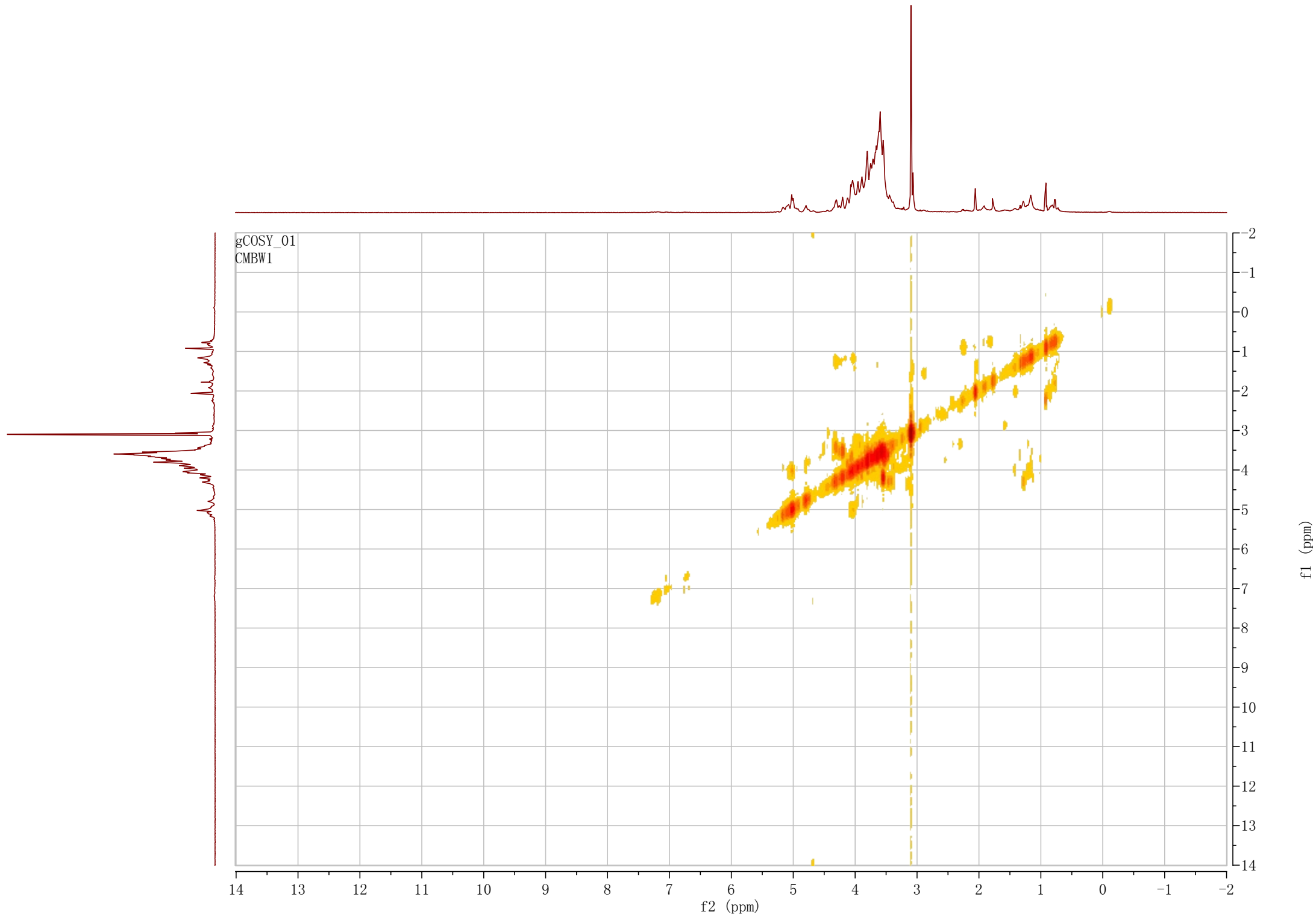

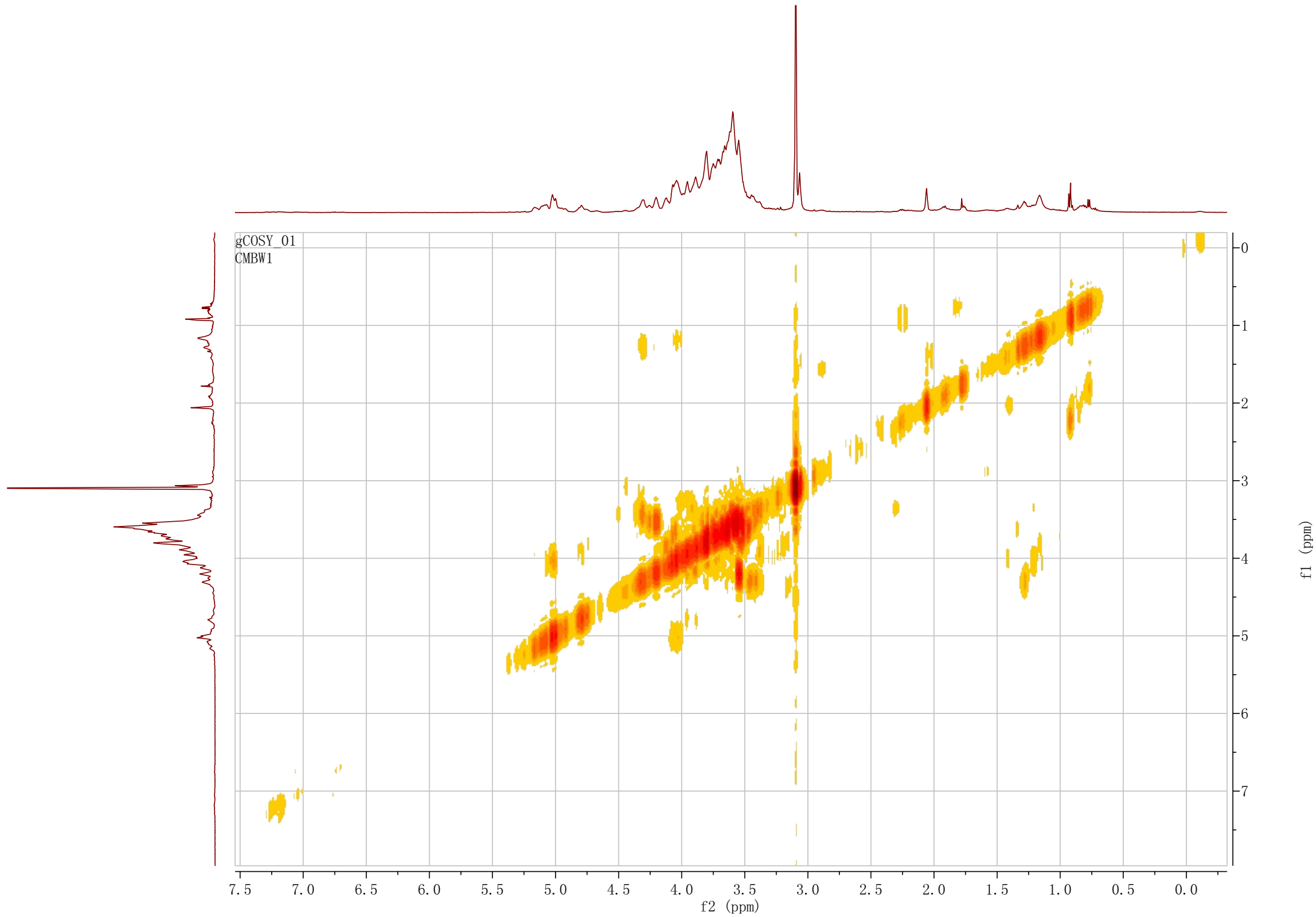

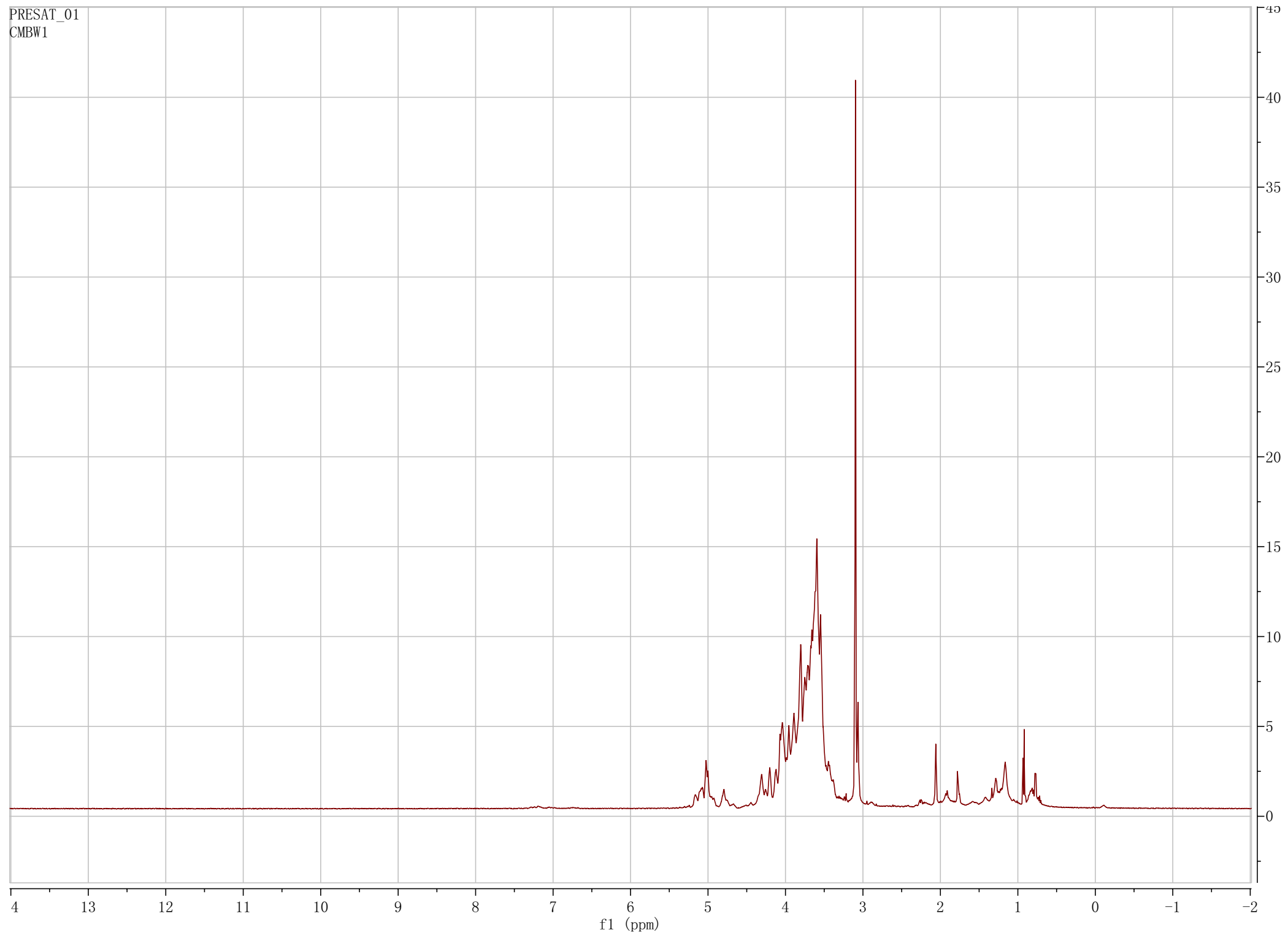

Supplement: Multimedia component 1 [file mmc1.pdf]

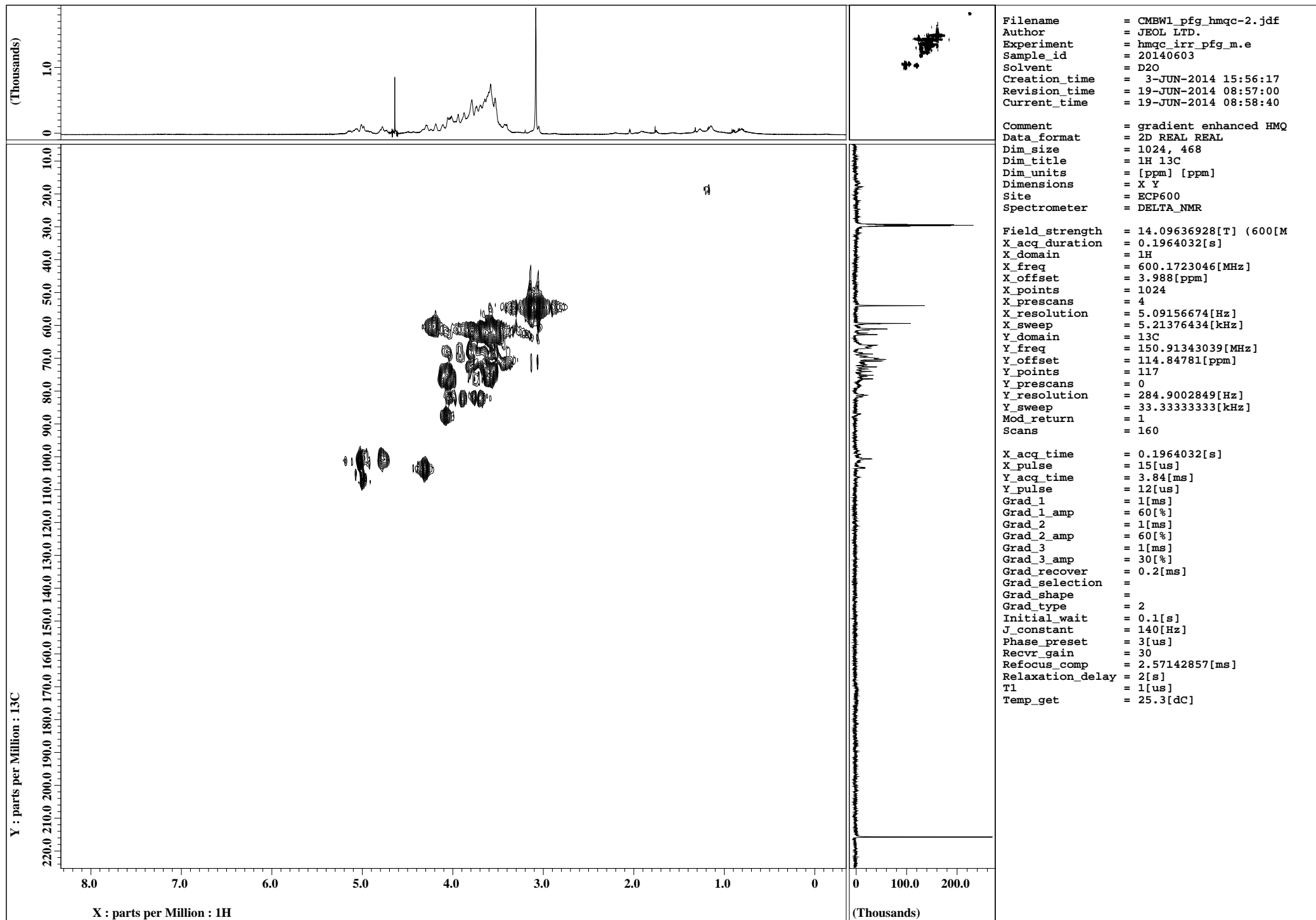

Supplement: Multimedia component 2 [file mmc2.pdf]

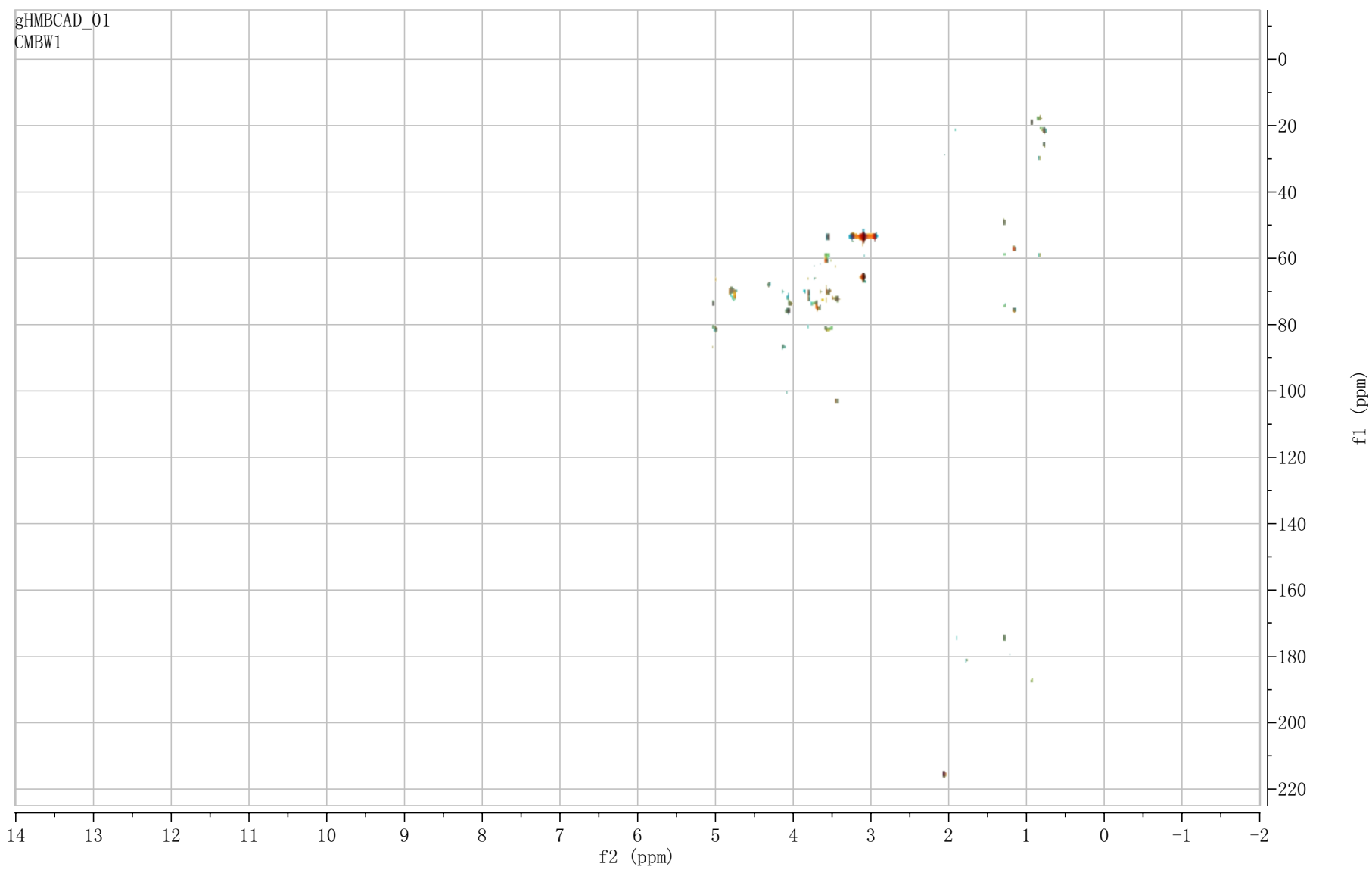

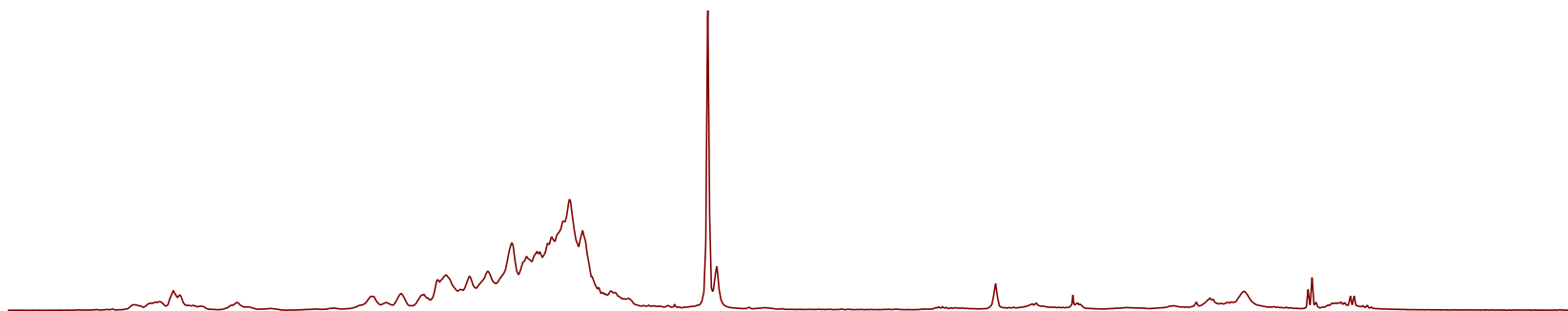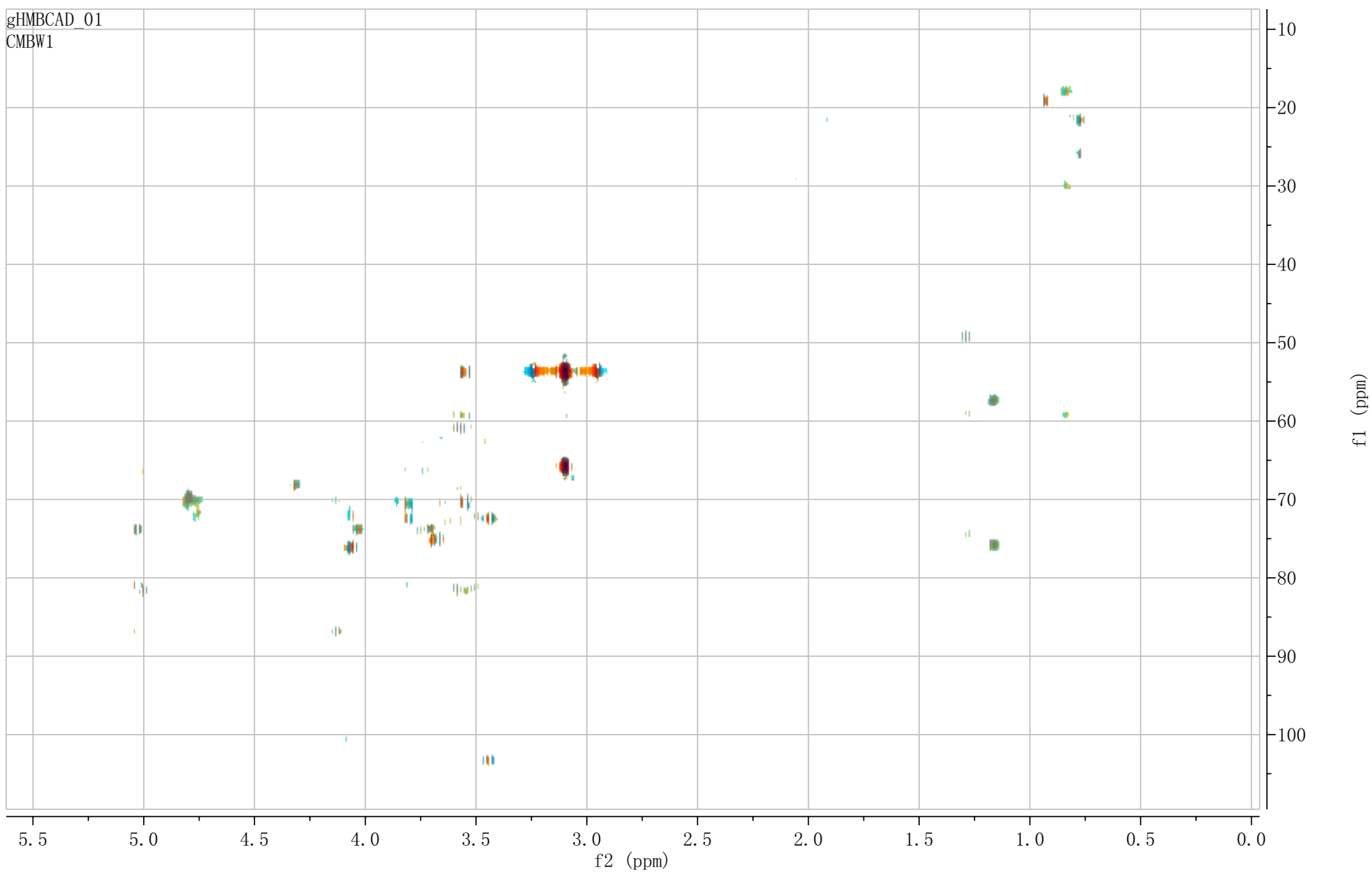

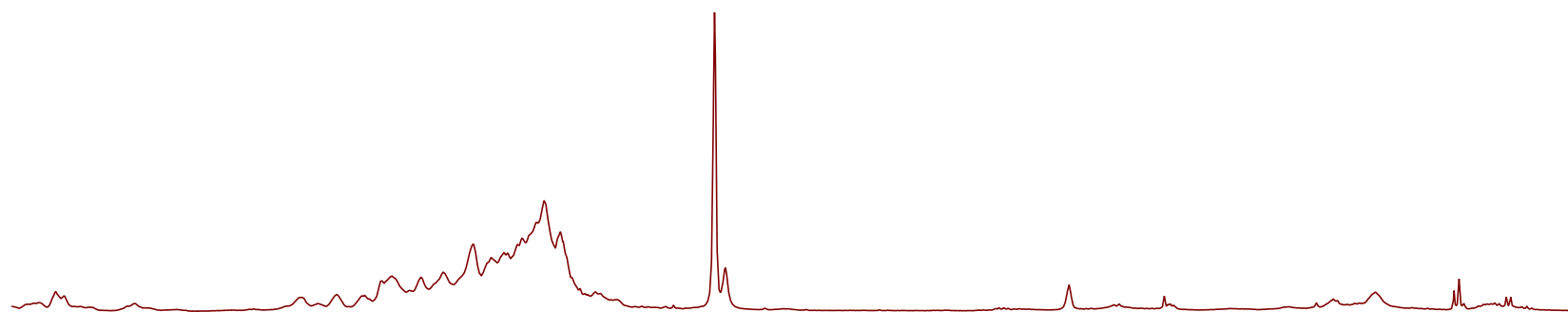

gHMBCAD\_01  
CMBW1

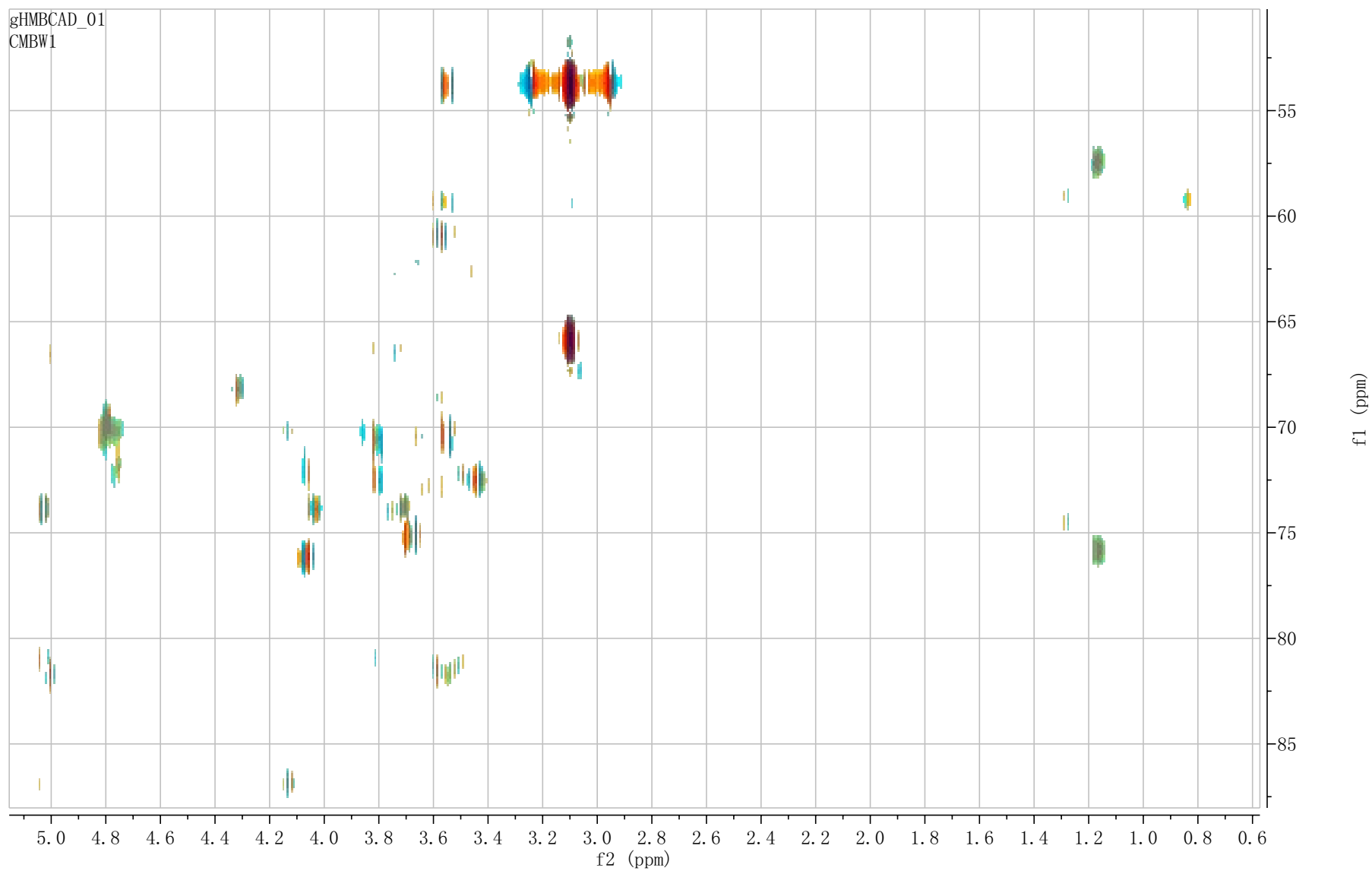

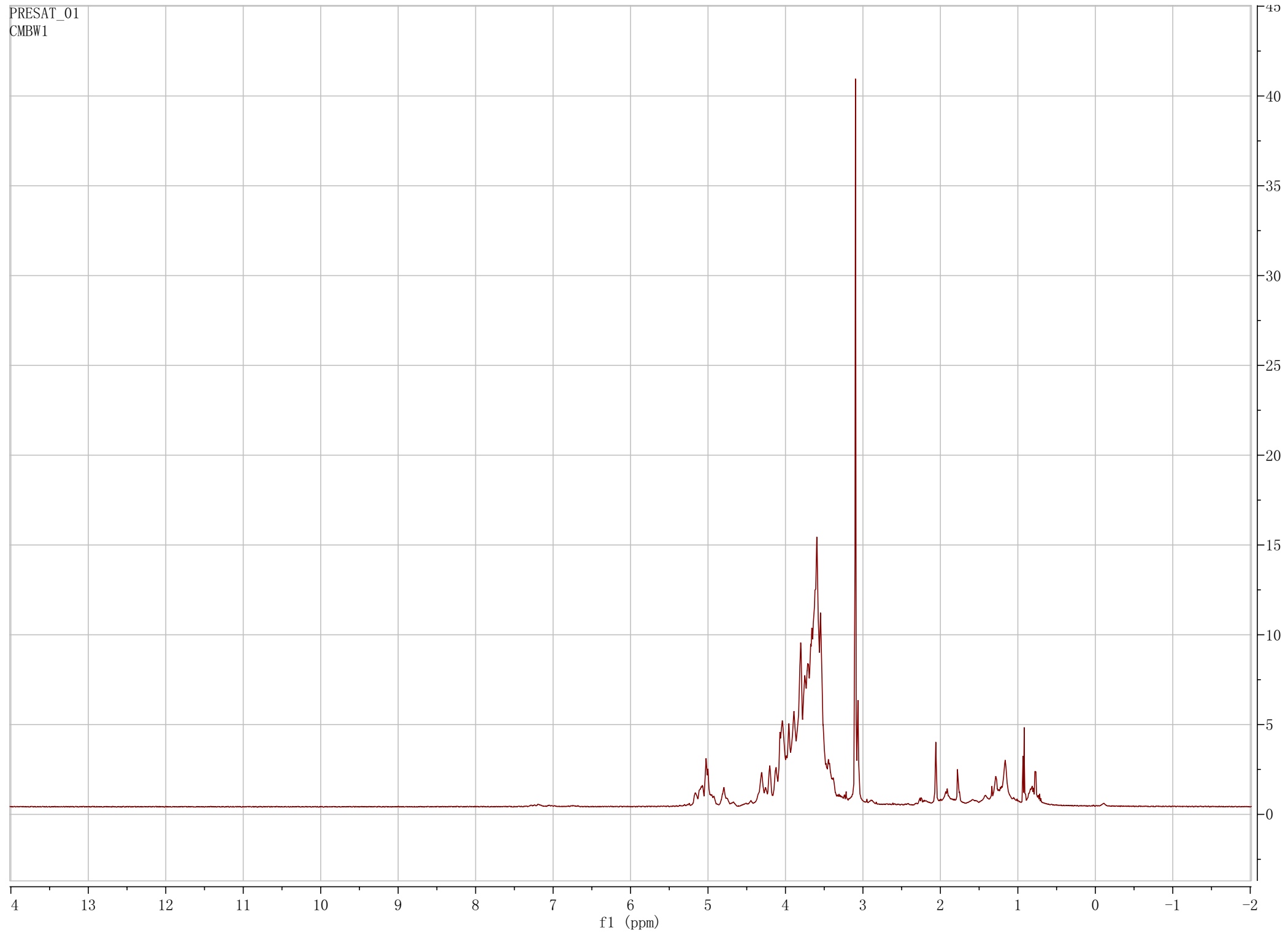

20140317  
Single Pulse with Broadband Decoupling

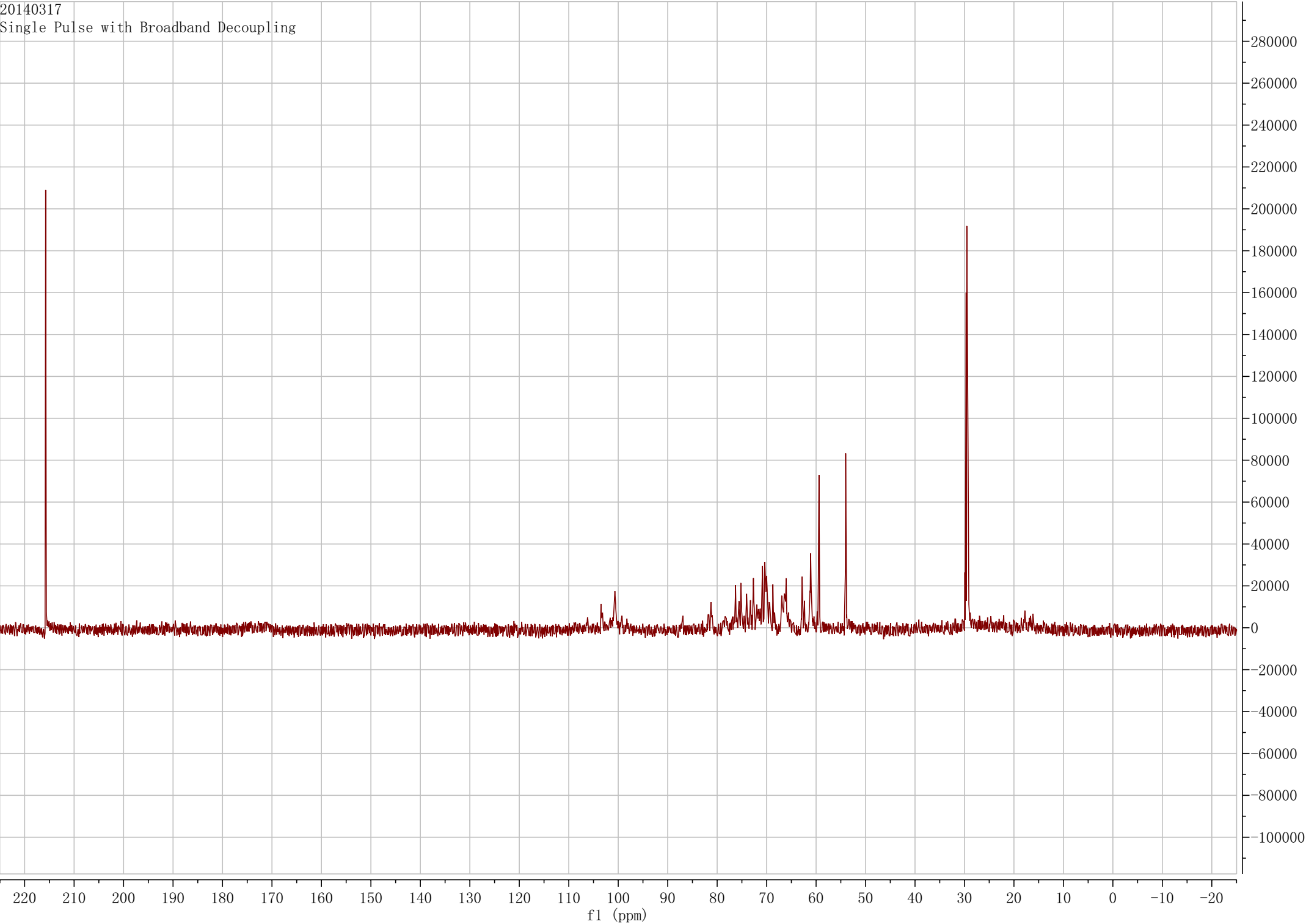

Supplement: Multimedia component 3 [file mmc3.pdf]

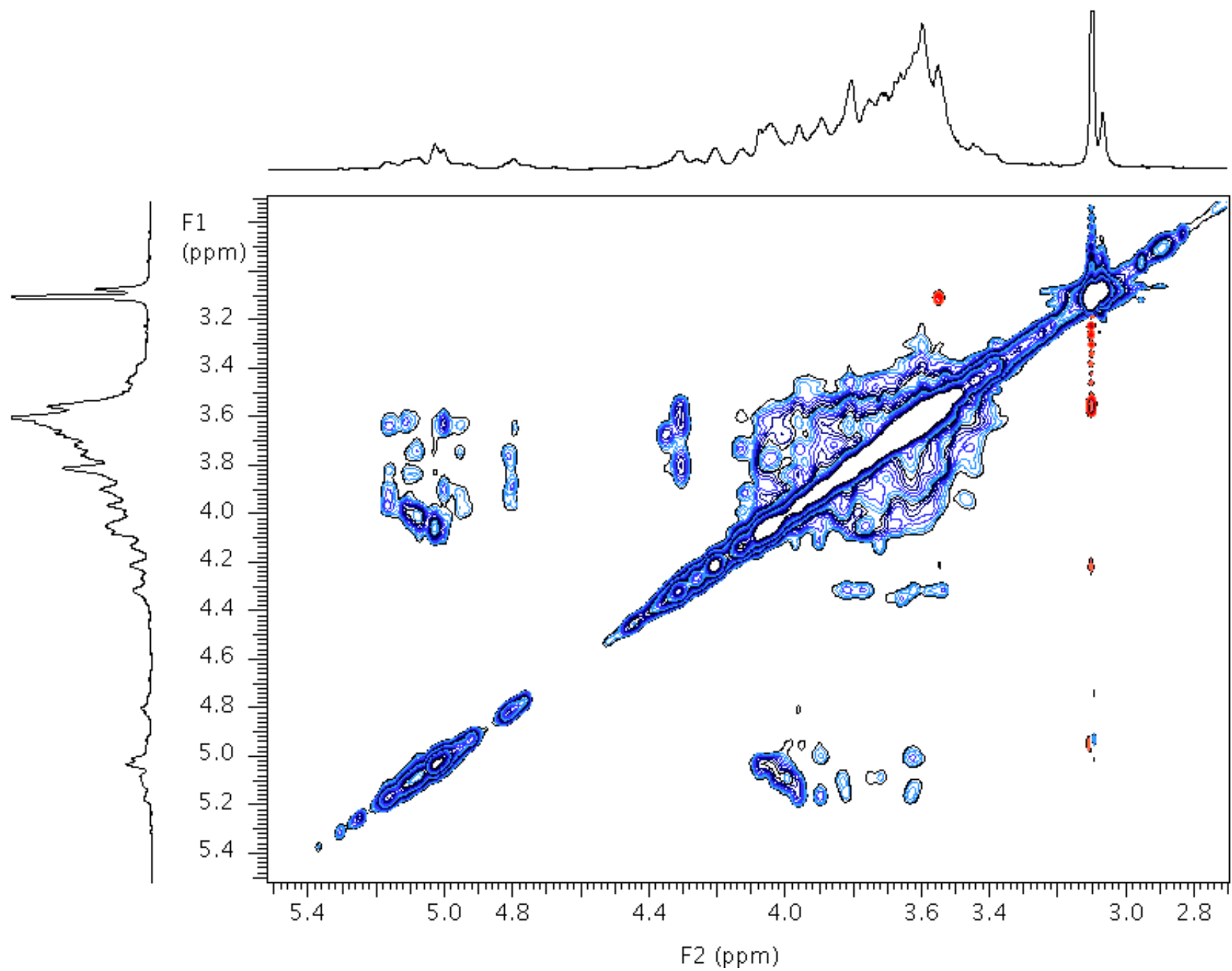

Supplement: Multimedia component 4 [file mmc4.pdf]

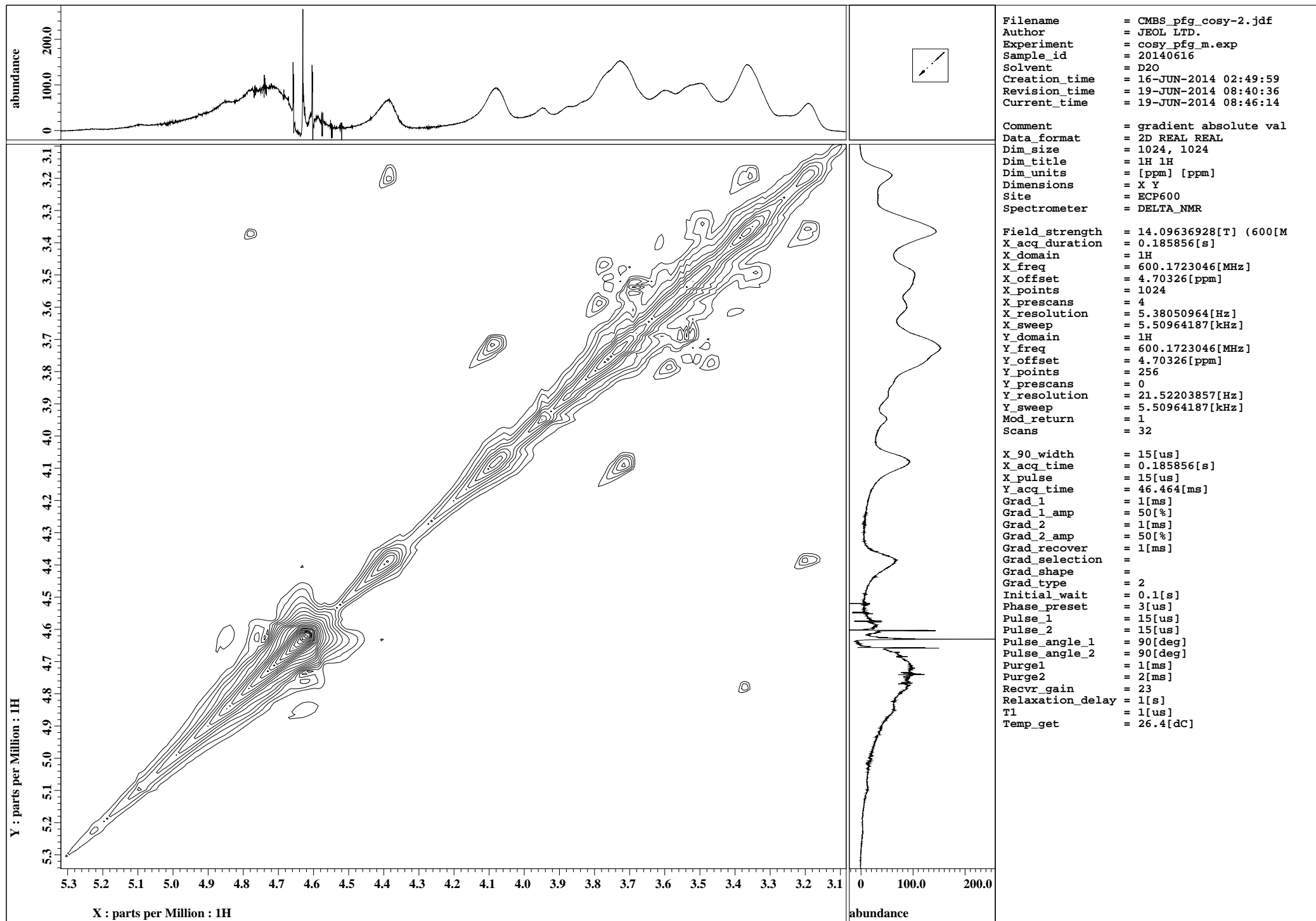

Supplement: Multimedia component 5 [file mmc5.pdf]

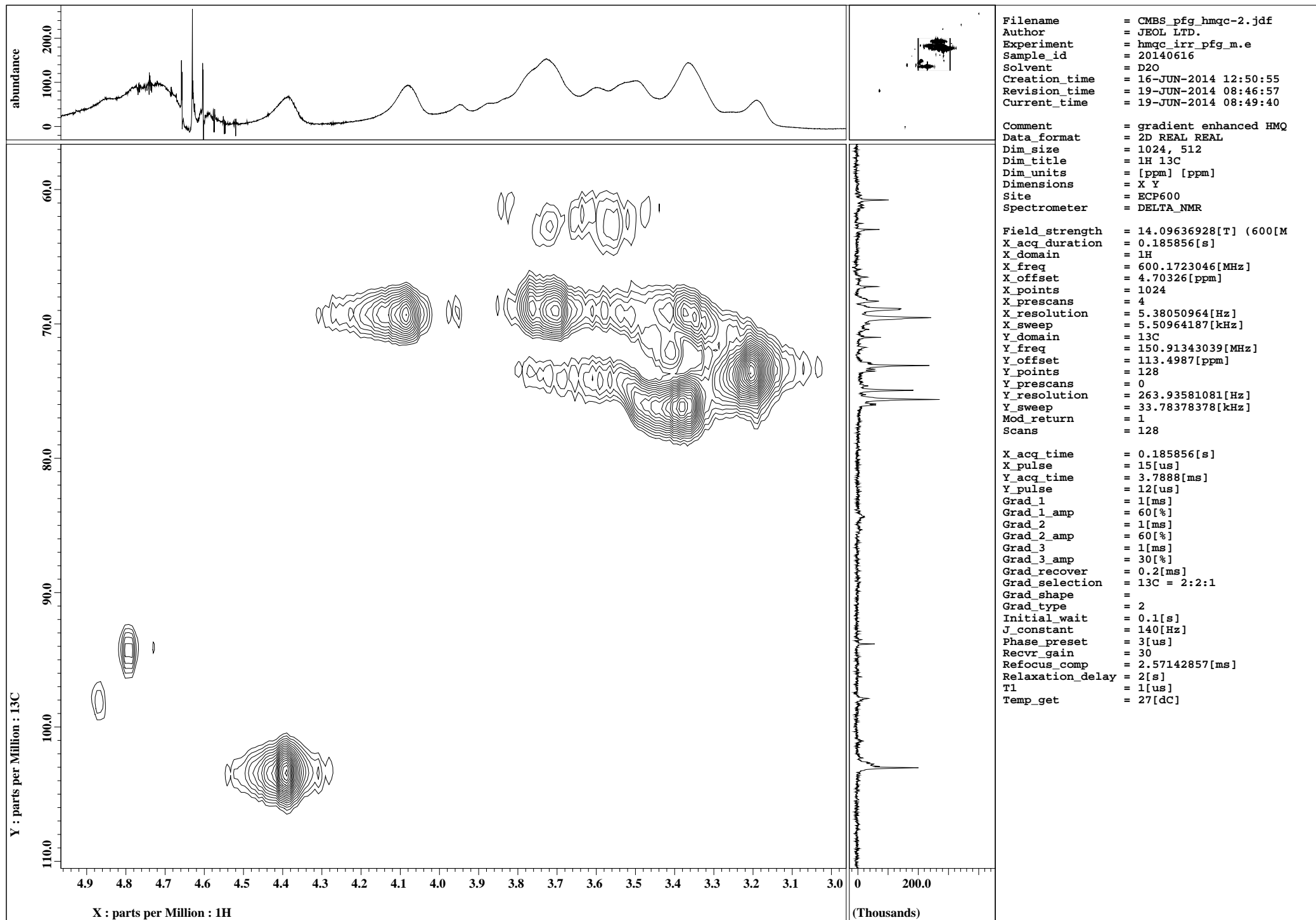

Supplement: Multimedia component 6 [file mmc6.pdf]

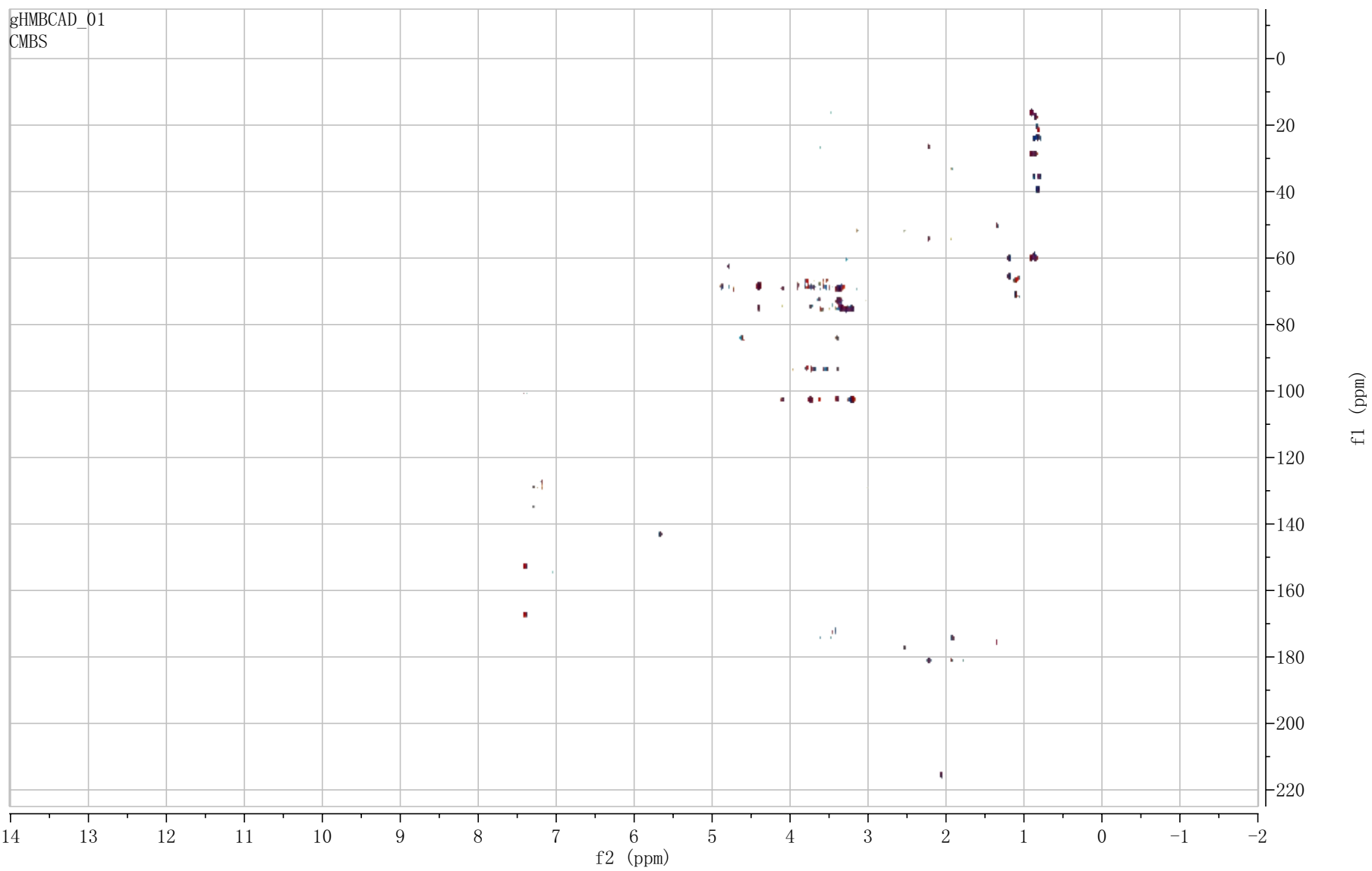

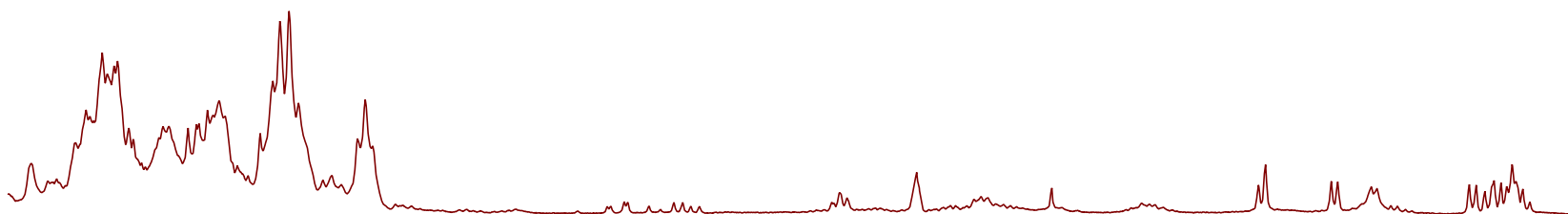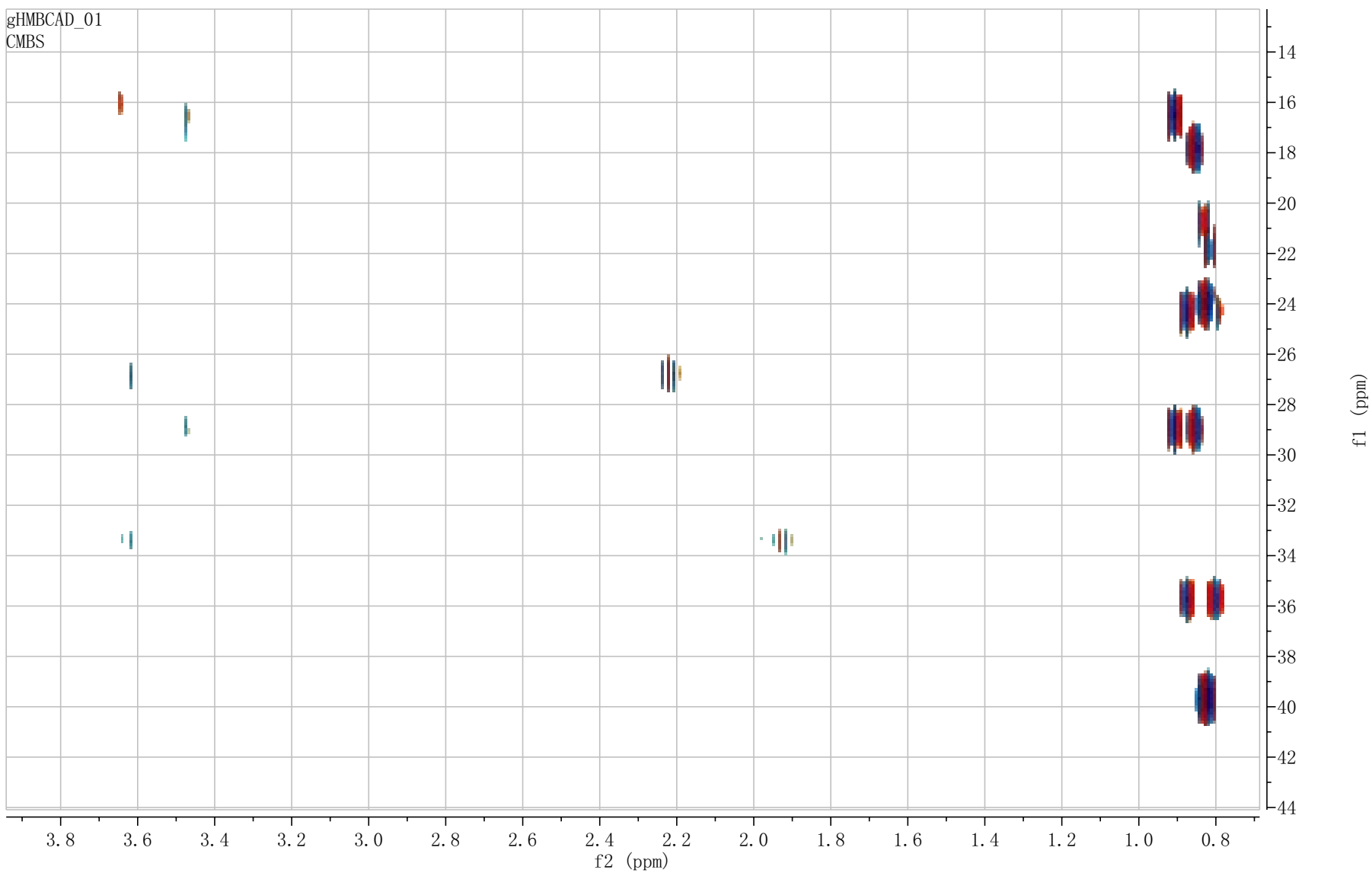

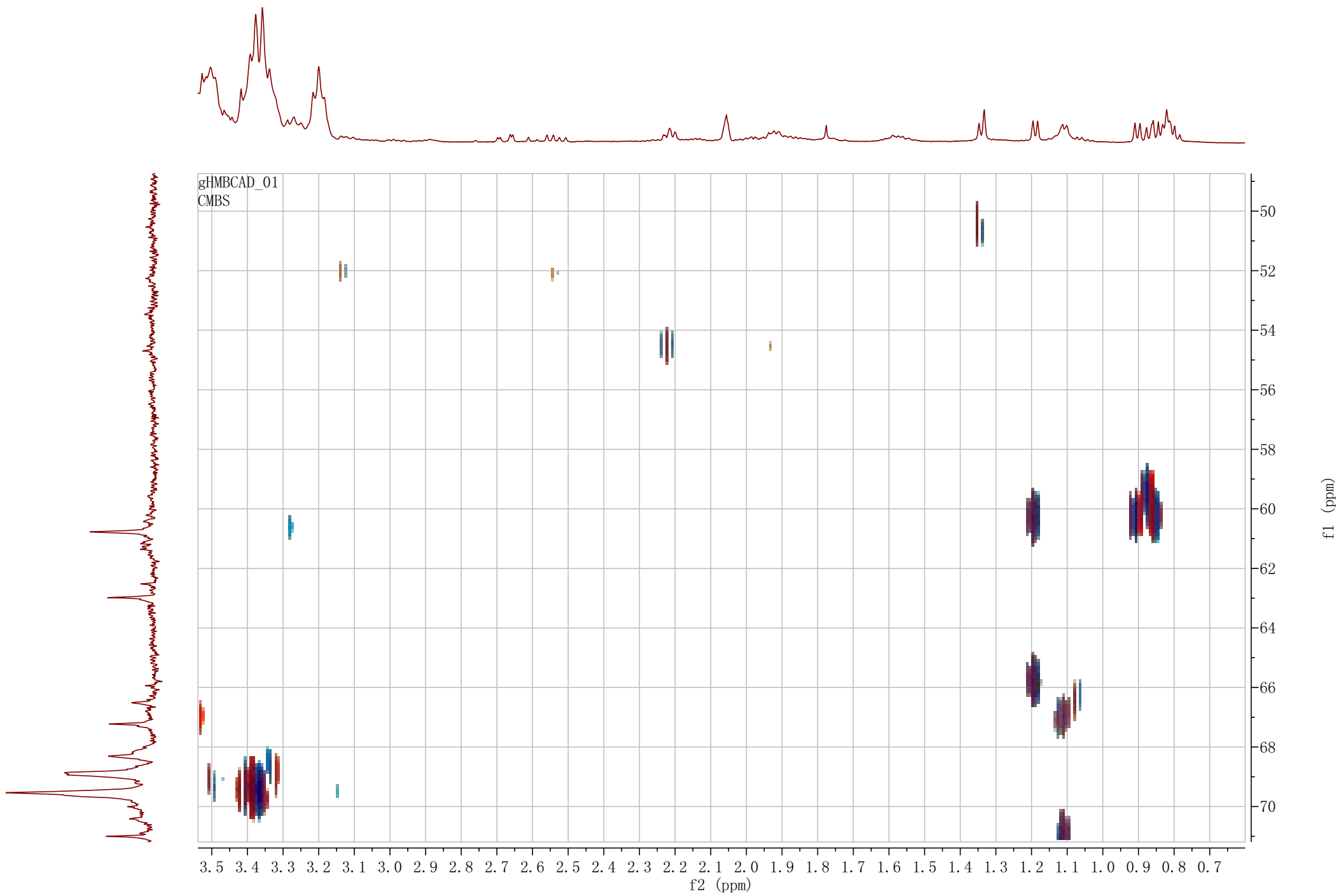

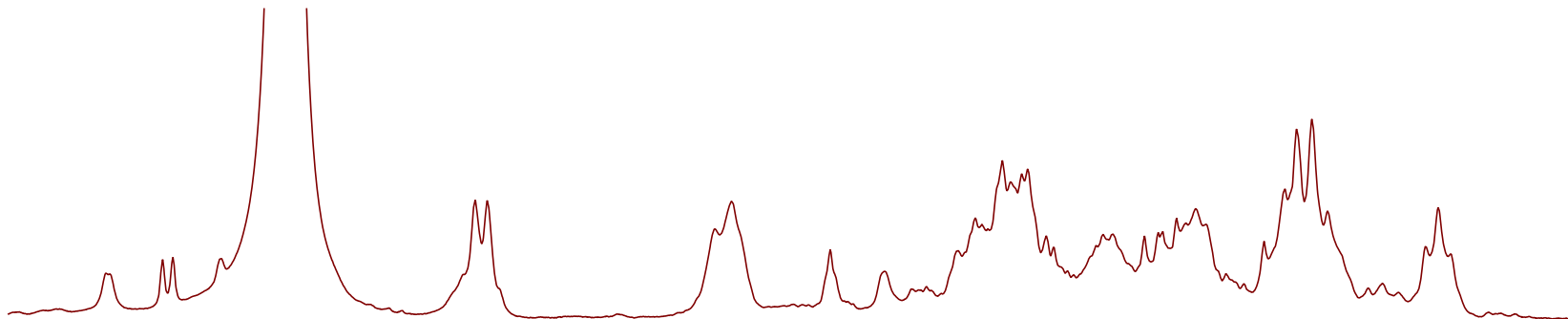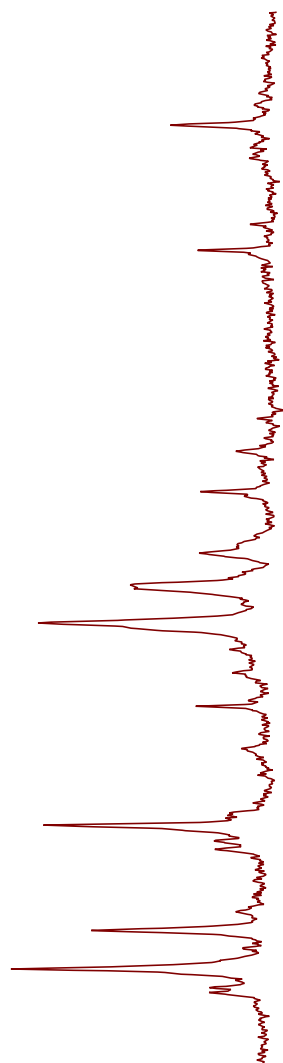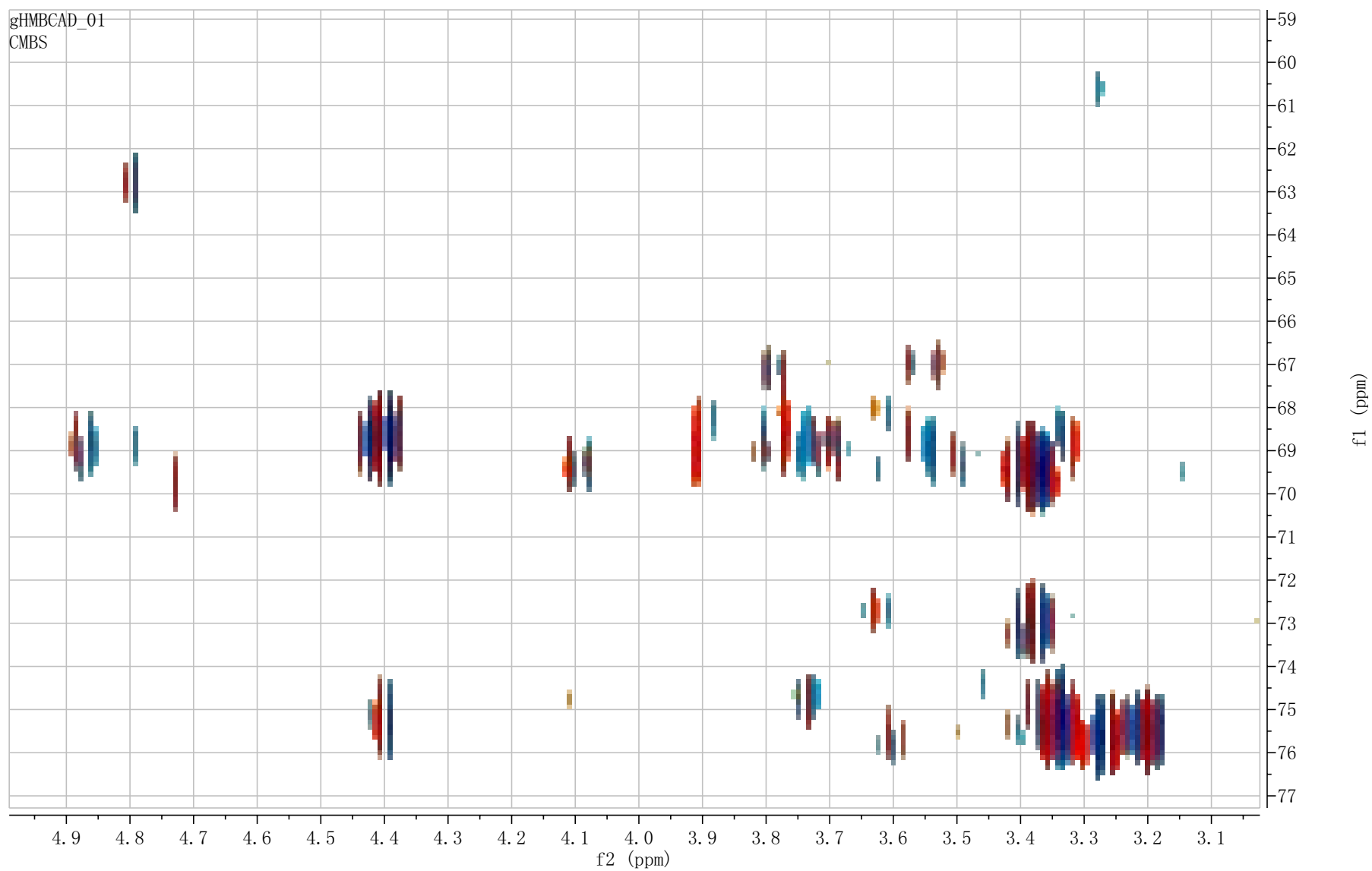

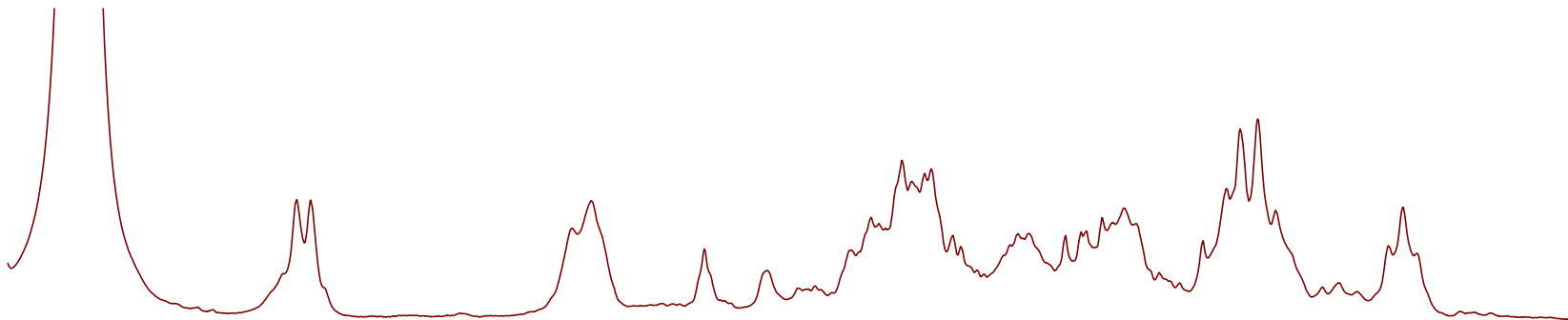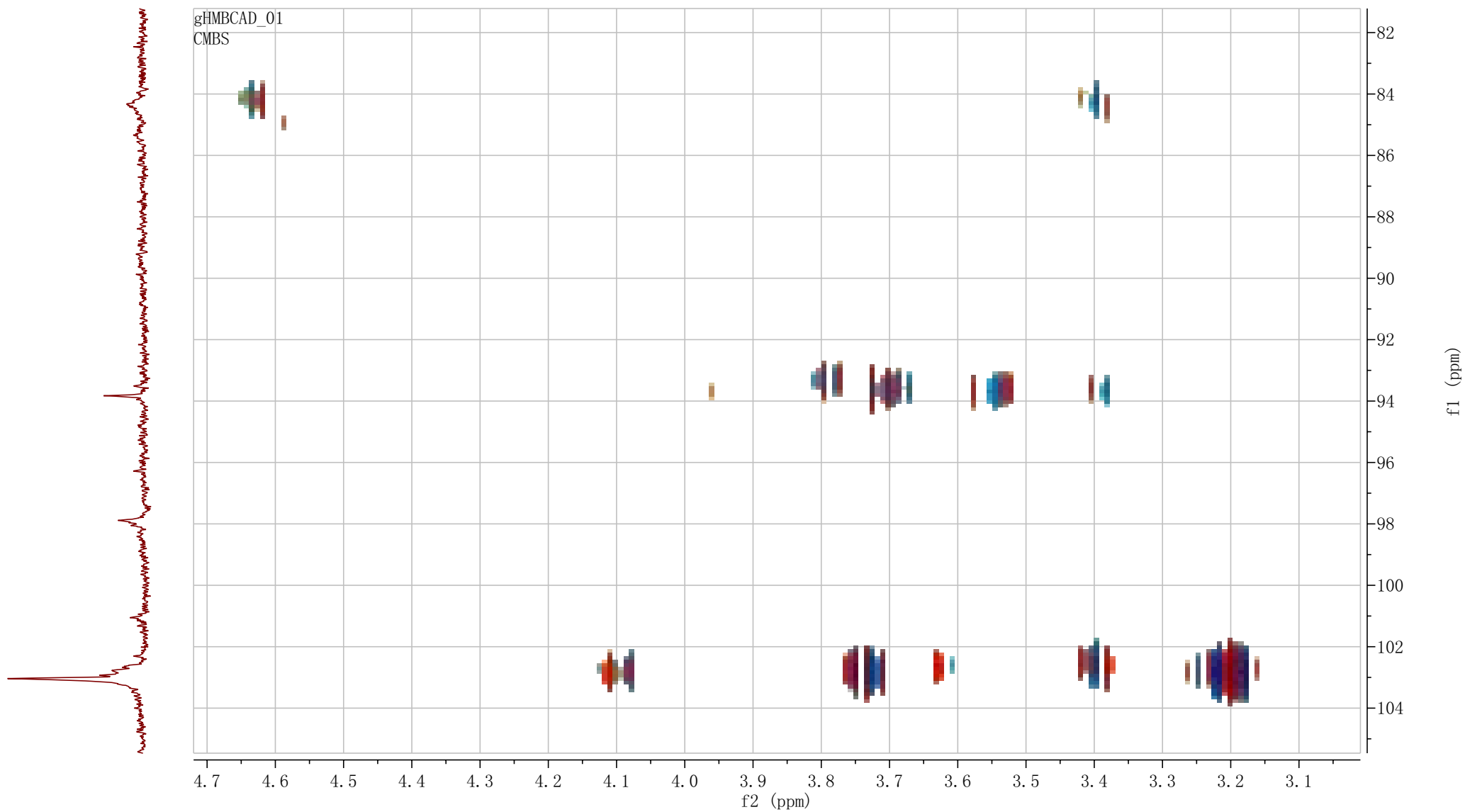

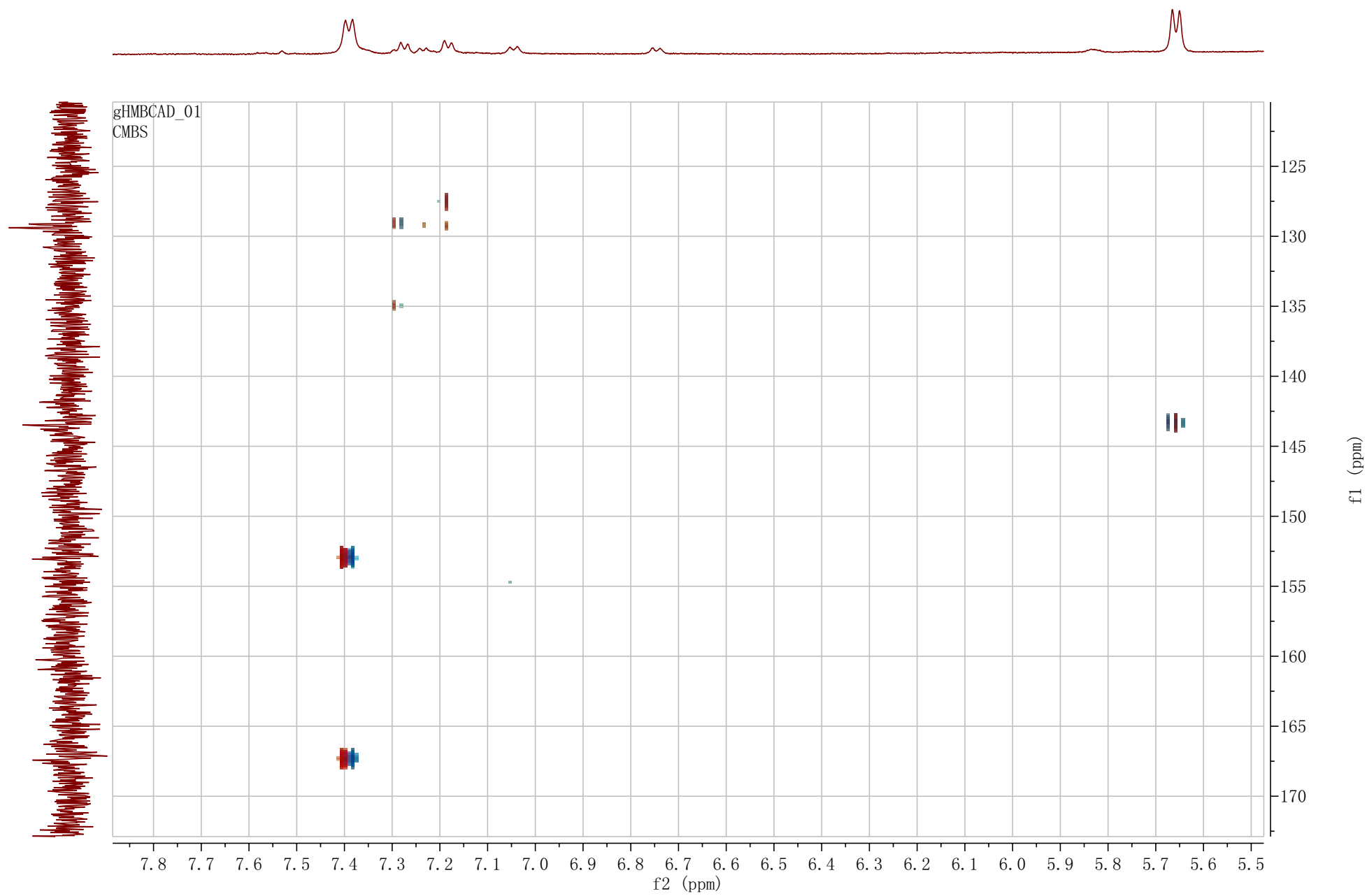

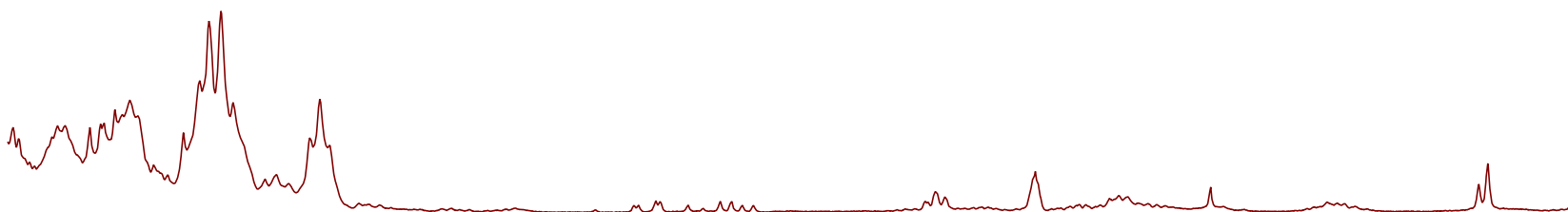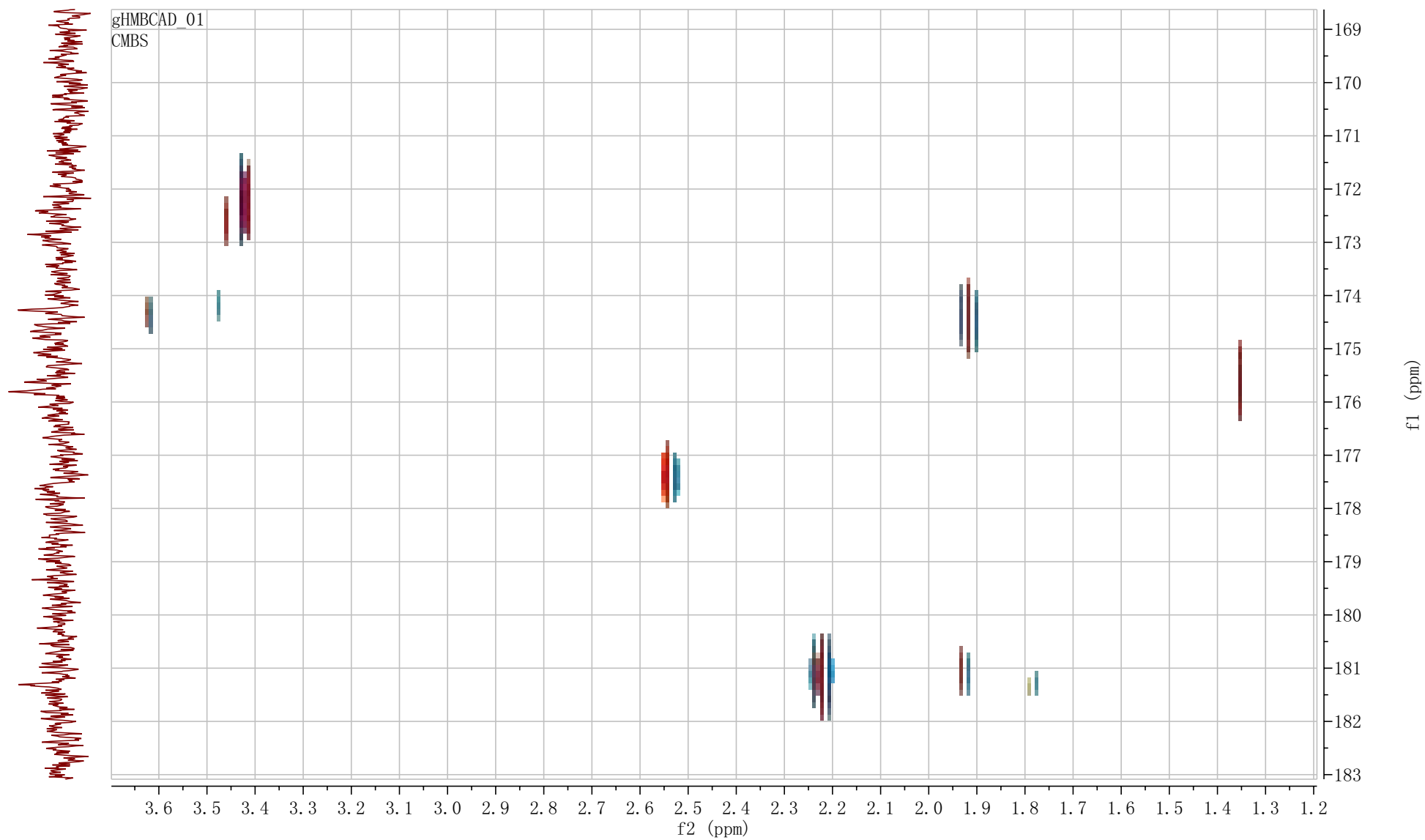

PROTON\_01  
CMBS

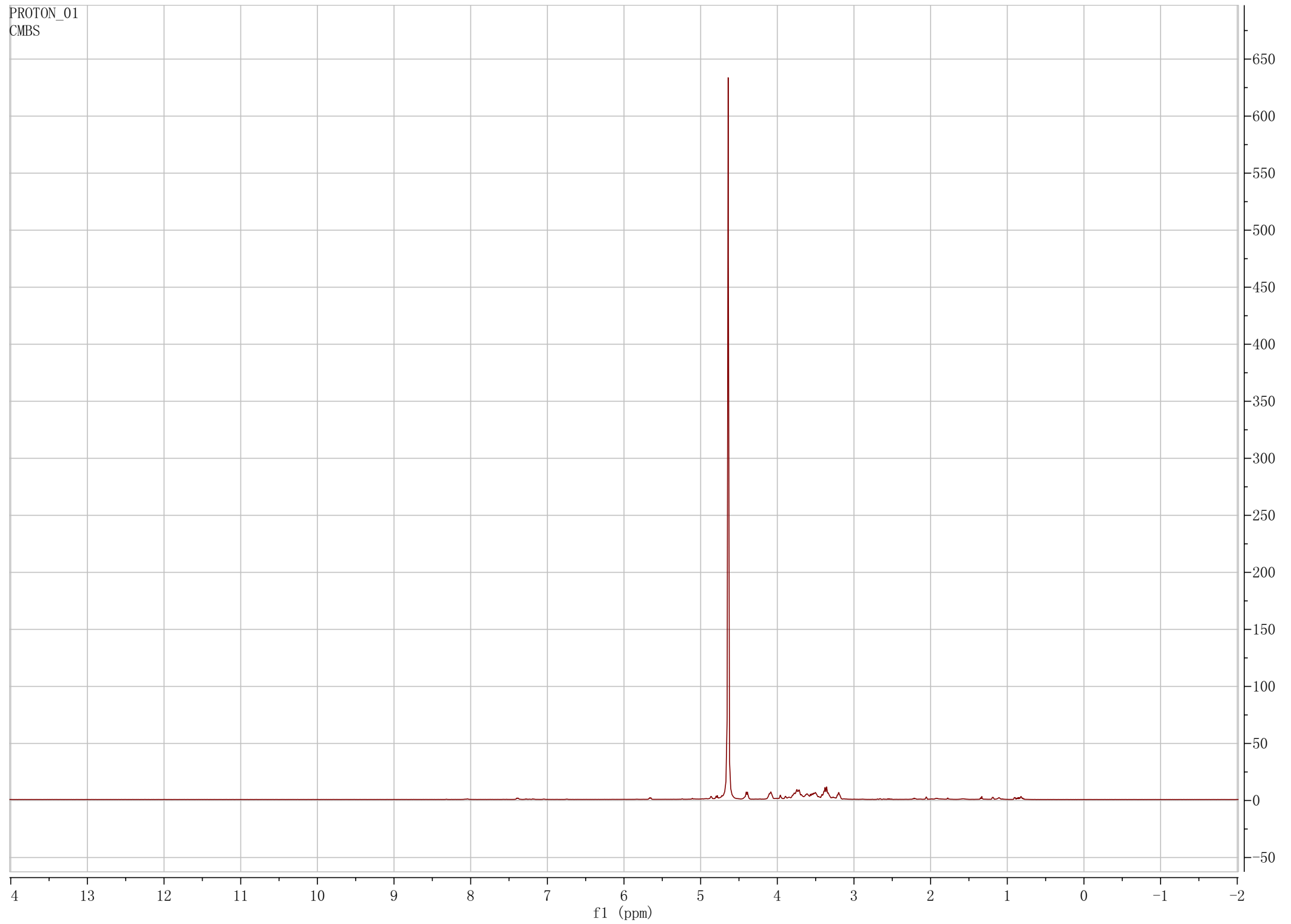

20140319  
Single Pulse with Broadband Decoupling

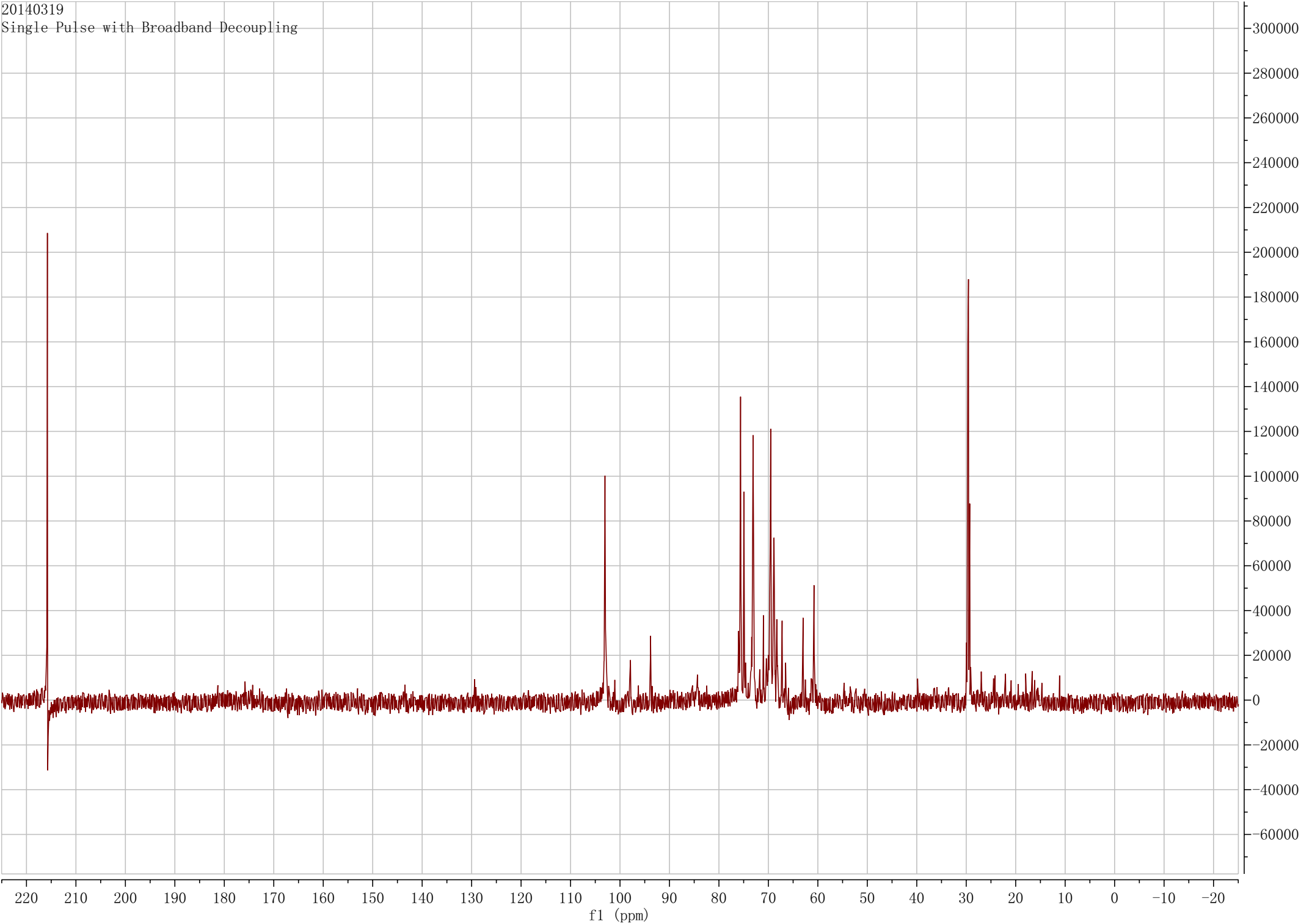

gHMBCAD\_01\_projection\_f1

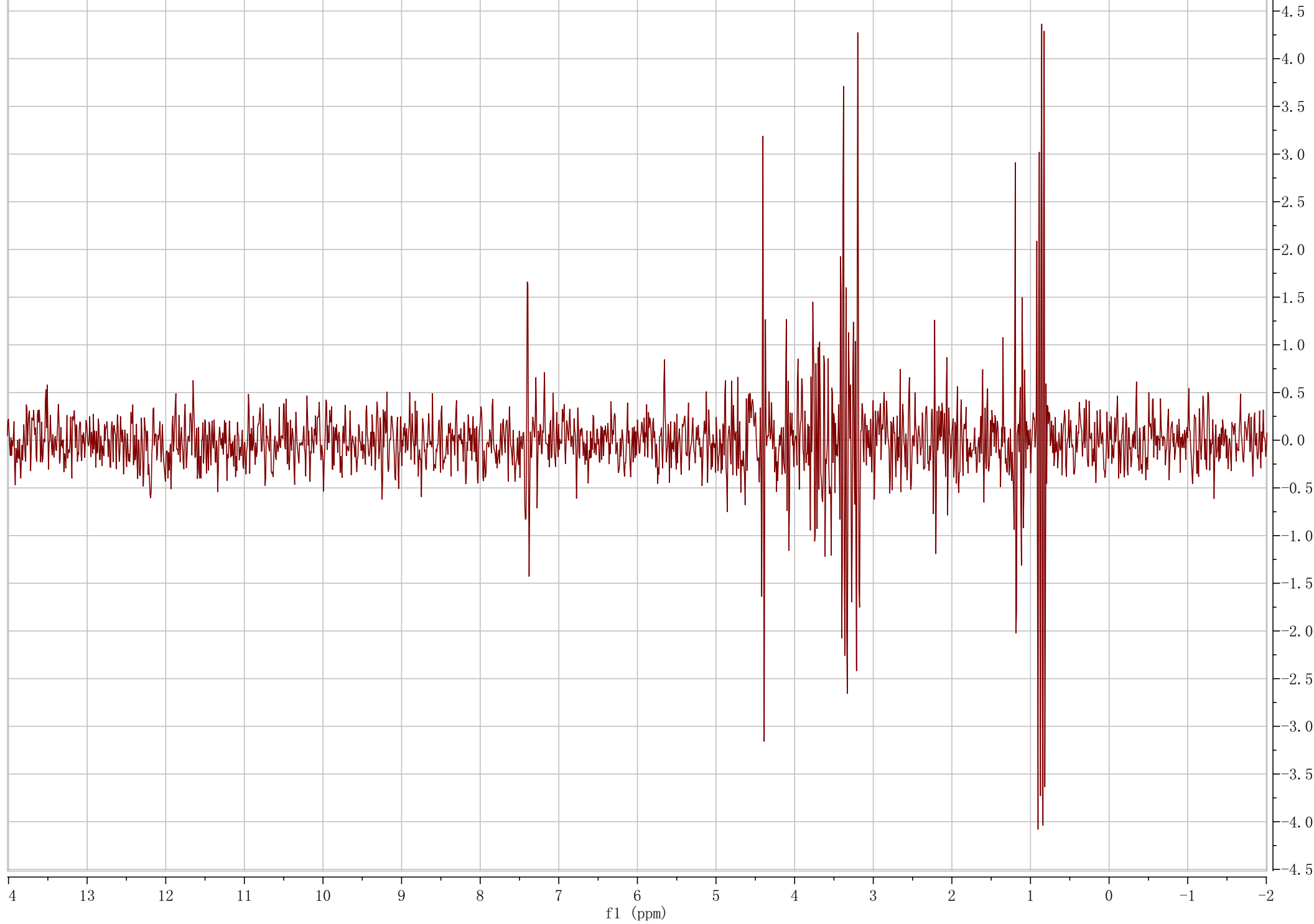

Supplement: Multimedia component 7 [file mmc7.pdf]

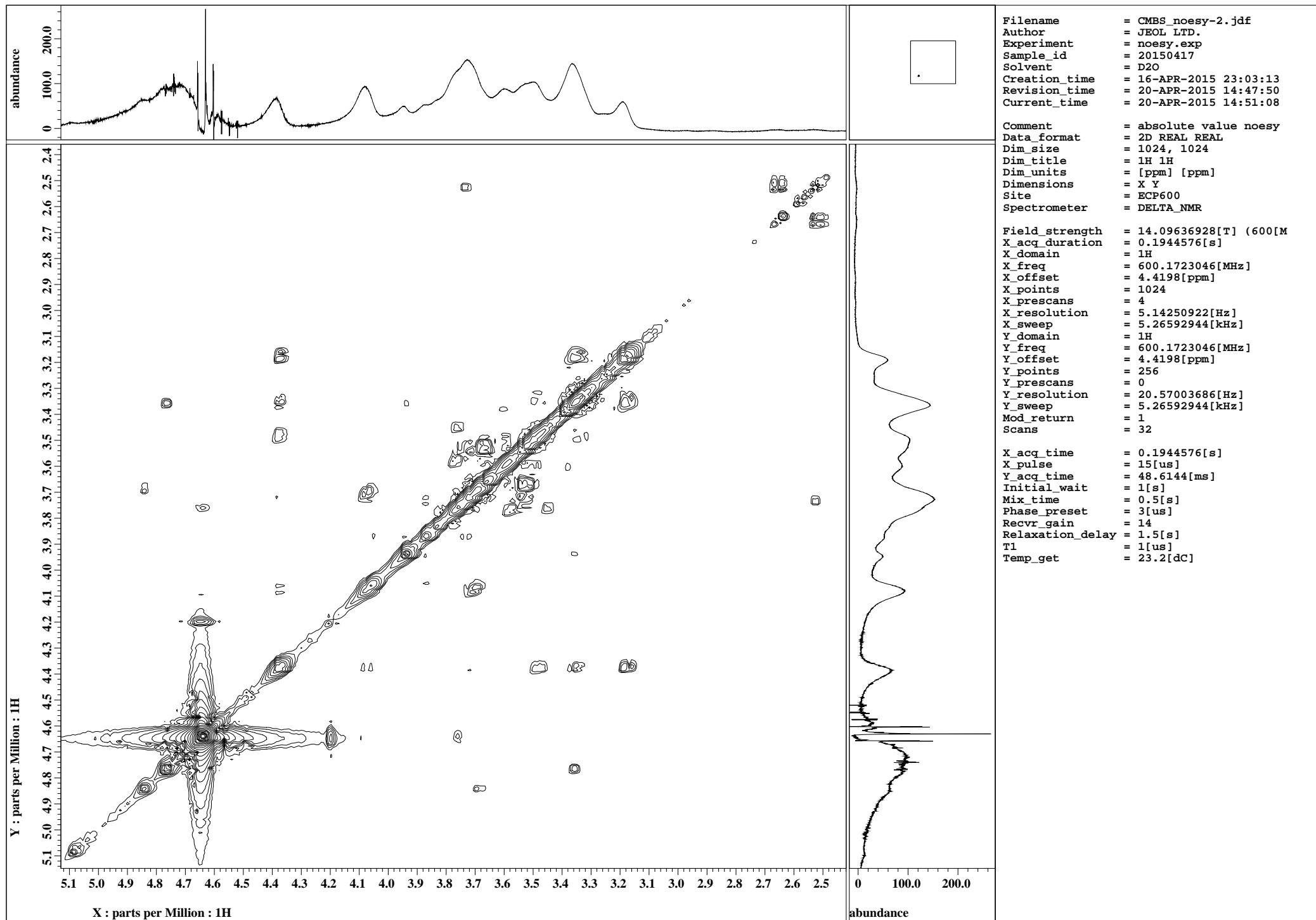

Supplement: Multimedia component 8 [file mmc8.pdf]

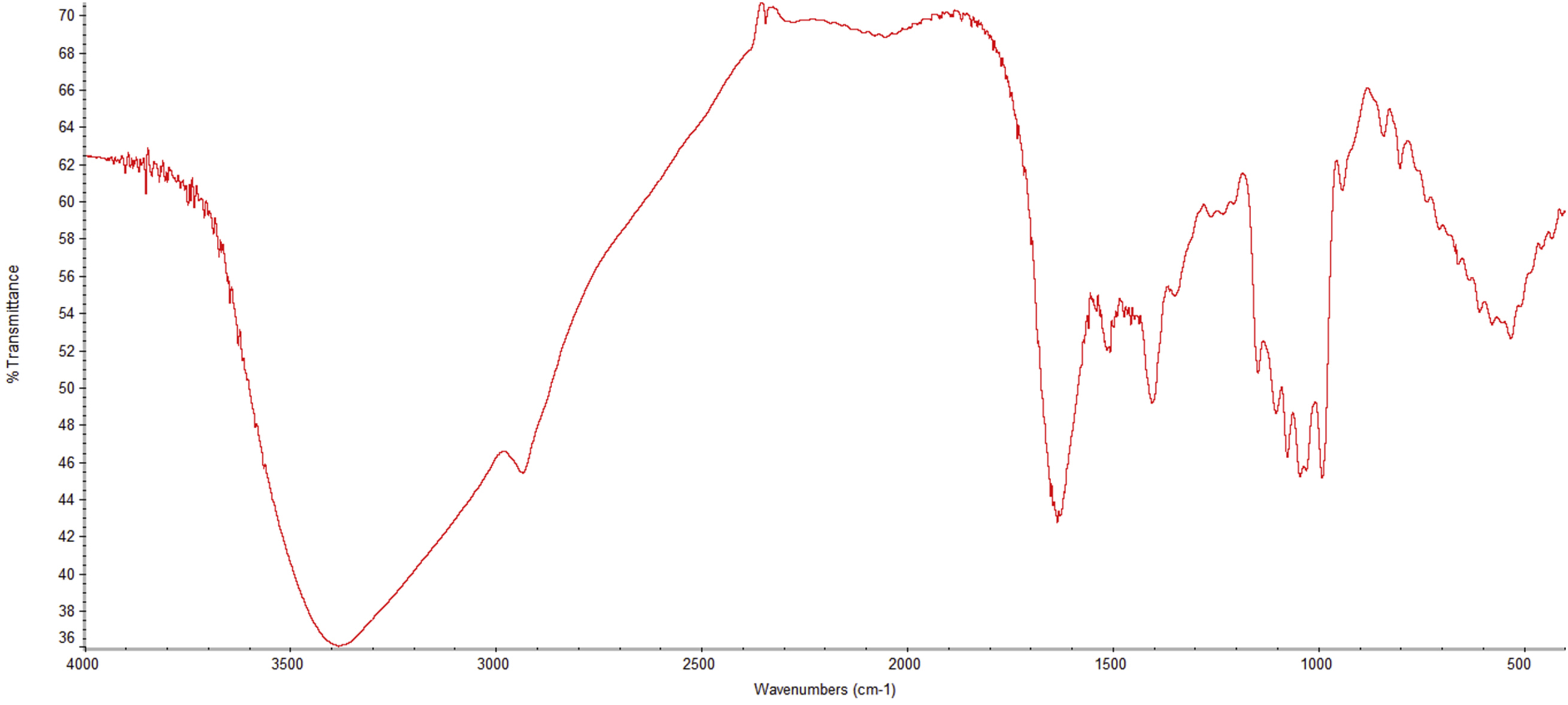

Supplement: Fig. S1 [file figs1.jpg]

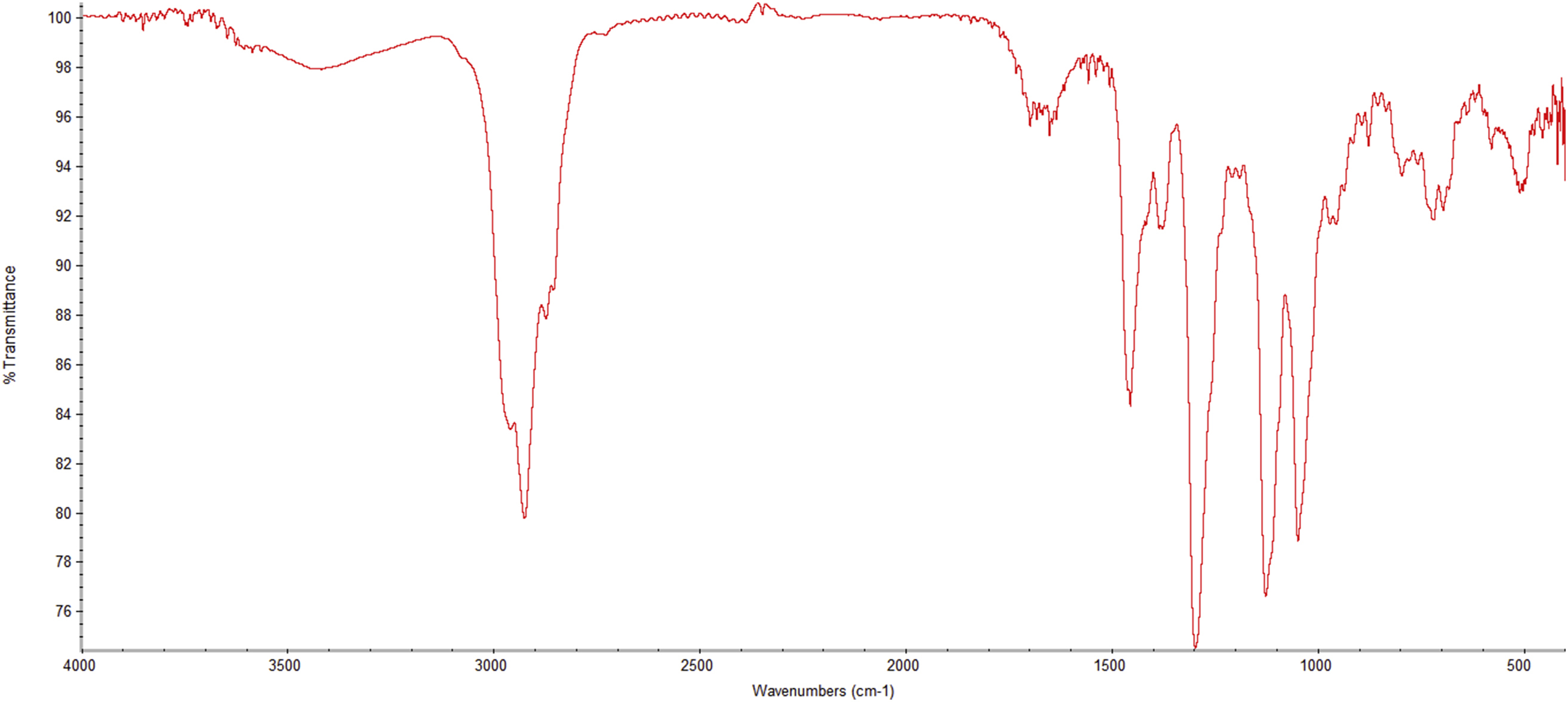

Supplement: Fig. S2 [file figs2.jpg]

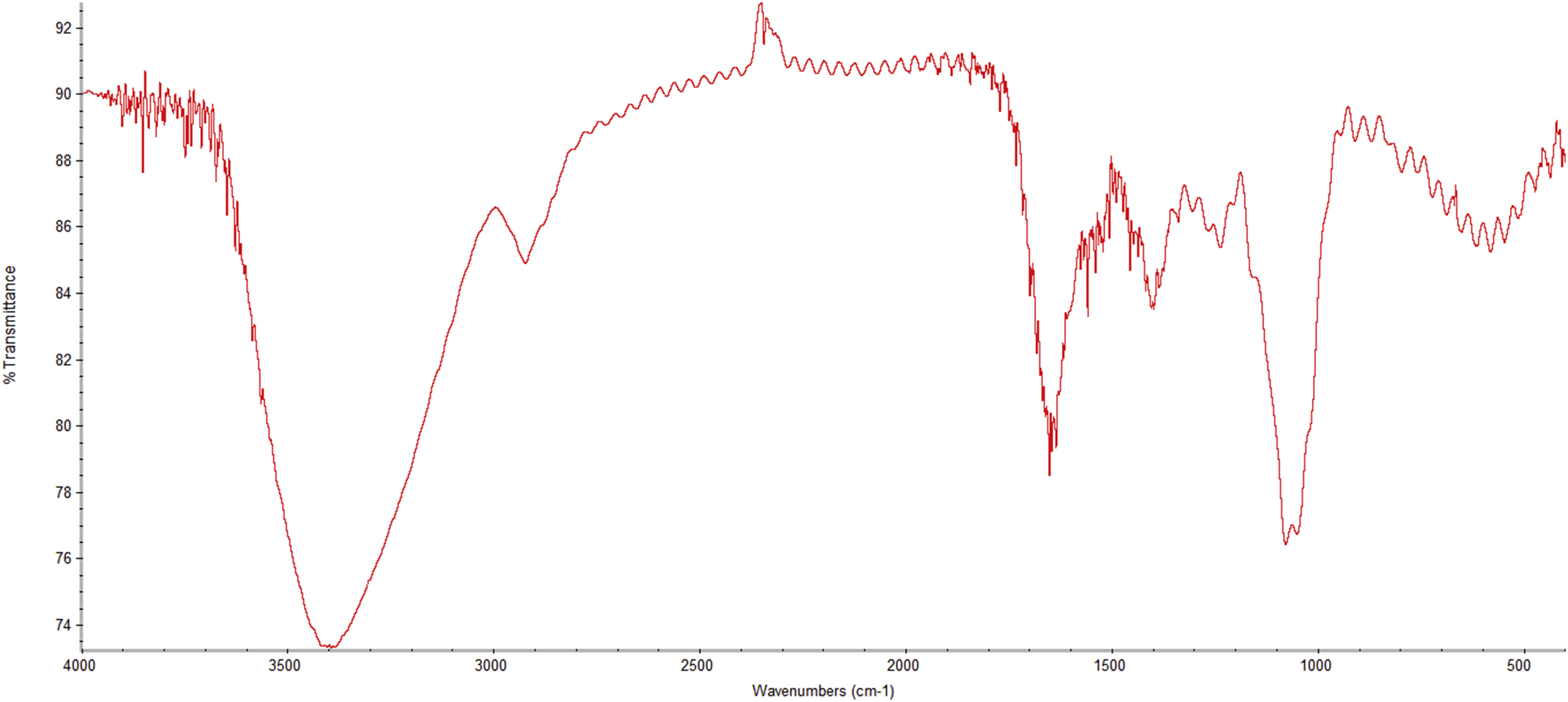

Supplement: Fig. S3 — 3 [file figs3.jpg]

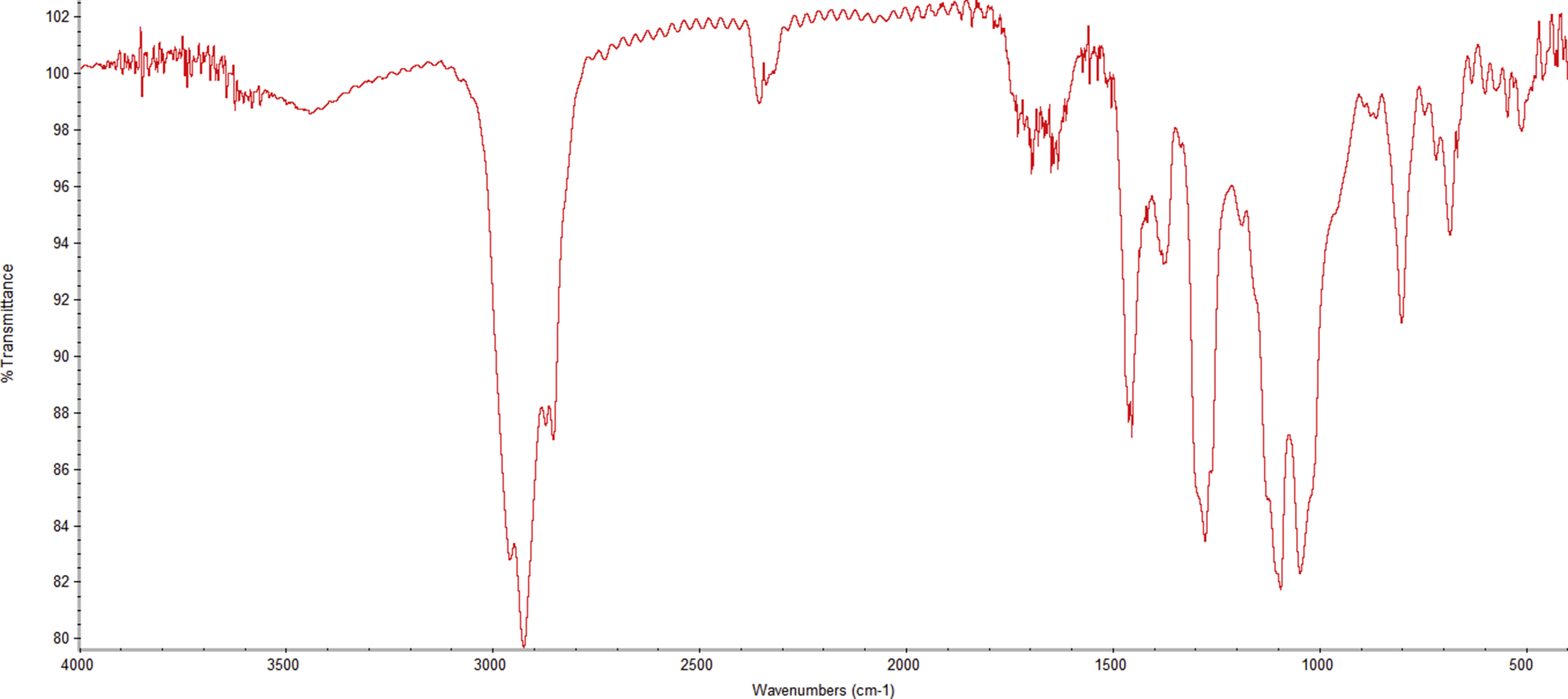

Supplement: Fig. S4 [file figs4.jpg]

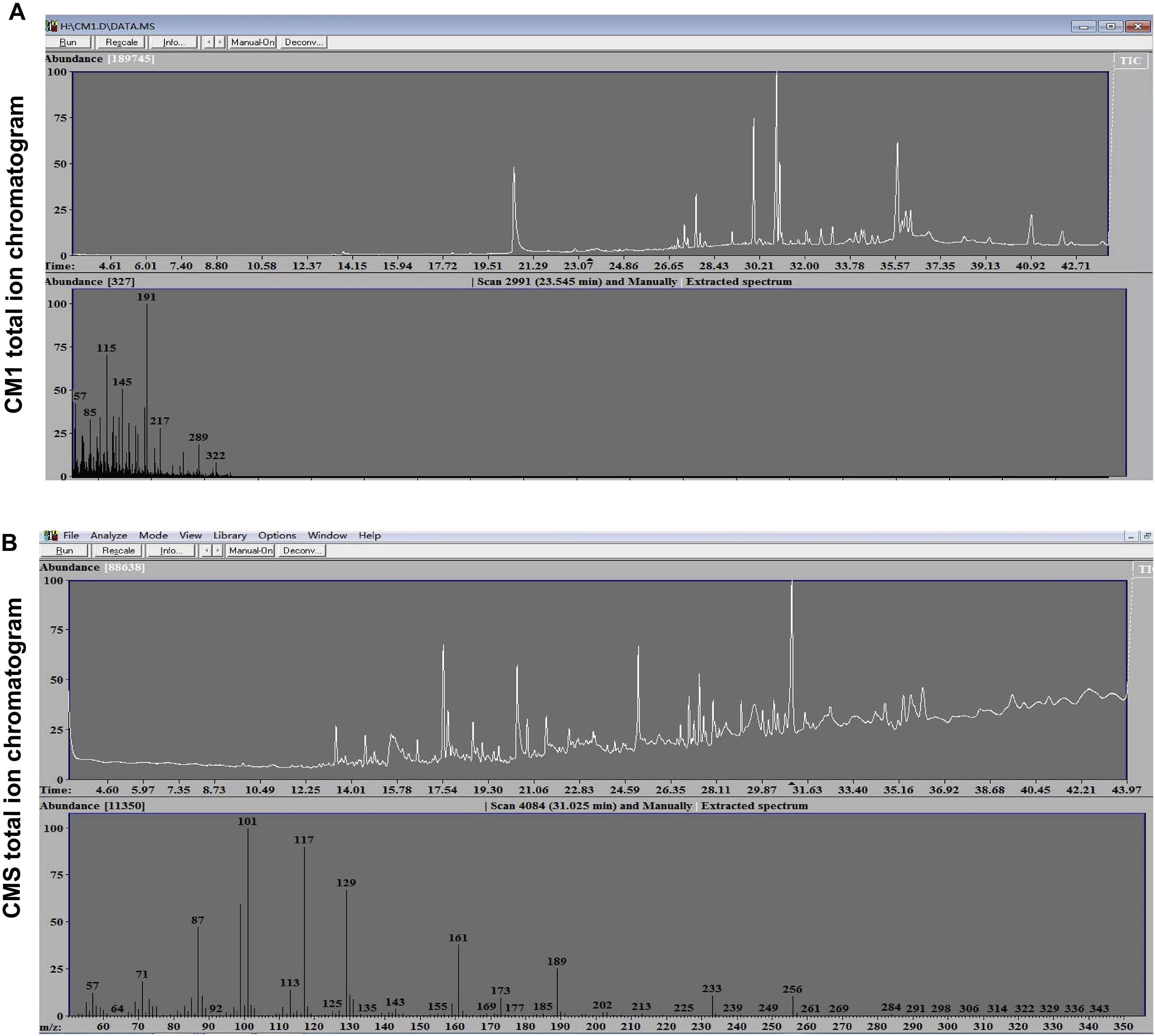

Supplement: Fig. S5 [file figs5.jpg]
